# Interaction of Iron II Complexes with B-DNA. Insights from Molecular Modeling, Spectroscopy, and Cellular Biology

**DOI:** 10.3389/fchem.2015.00067

**Published:** 2015-12-18

**Authors:** Hugo Gattuso, Thibaut Duchanois, Vanessa Besancenot, Claire Barbieux, Xavier Assfeld, Philippe Becuwe, Philippe C. Gros, Stephanie Grandemange, Antonio Monari

**Affiliations:** ^1^Université de Lorraine – Nancy and Centre National de la Recherche Scientifique, Theory-Simulation-Modeling, SRSMCNancy, France; ^2^Université de Lorraine – Nancy and Centre National de la Recherche Scientifique, Hécrin, SRSMCNancy, France; ^3^Université de Lorraine – Nancy and Centre National de la Recherche Scientifique, CRANNancy, France

**Keywords:** molecular modeling, iron complexes, DNA sensitization, non-covalent interactions, cell viability

## Abstract

We report the characterization of the interaction between B-DNA and three terpyridin iron II complexes. Relatively long time-scale molecular dynamics (MD) is used in order to characterize the stable interaction modes. By means of molecular modeling and UV-vis spectroscopy, we prove that they may lead to stable interactions with the DNA duplex. Furthermore, the presence of larger π-conjugated moieties also leads to the appearance of intercalation binding mode. Non-covalent stabilizing interactions between the iron complexes and the DNA are also characterized and evidenced by the analysis of the gradient of the electronic density. Finally, the structural deformations induced on the DNA in the different binding modes are also evidenced. The synthesis and chemical characterization of the three complexes is reported, as well as their absorption spectra in presence of DNA duplexes to prove the interaction with DNA. Finally, their effects on human cell cultures have also been evidenced to further enlighten their biological effects.

## Introduction

Since the celebrated discovery of cis-platin in the seventies (Rosenberg and VanCamp, 1970), the research of novel molecules interacting with DNA is a very active field (Reedijk, 2009; Rescifina et al., 2014). Indeed, the selective interaction between drugs and DNA may lead to extremely significant therapeutic activity. DNA interacting drugs are nowadays currently used in the treatment of many diseases ranging from cancer, to chronic inflammation, as well as in the fight against resistant bacterial or viral infections (Darren et al., 2009; Alketa et al., 2013; Nahid and Moghadam, 2013). The therapeutic activity of DNA interactors can be straightforwardly related to the crucial biological role played by the nucleic acids (Dumont and Monari, 2015). Indeed, the production of permanent damages to DNA, or on the contrary the stabilization of the DNA duplex and the consequent inhibition of replication, may usually lead to cellular apoptosis (Florea and Büsselberg, 2011). However, especially in anticancer therapy, the development of new drugs minimizing the unwanted side effects of the commercial ones is still most sought.

If cis-platin interacts with DNA by covalently bonding the duplex's backbone, in the last years a considerable interest has been devoted to non-covalent DNA bindings (sensitizers) (Erkkila et al., 1999; Nelson et al., 2007). Different classes of endogenous and exogenous molecules may exhibit specific interaction with the DNA, and hence be characterized by a different biological activity. DNA/drugs adducts are known for both small organic molecules, such as aryl ketones, and relatively large organometallic compounds, such as copper (Rajendiran et al., 2008; Kellet et al., 2011; Larragy et al., 2015; Molphy et al., 2015) or ruthenium polypyrimidines or polyphenantrolines (Very et al., 2012; Chantzis et al., 2014; Huang et al., 2014). Recently the interaction of Iridium complexes with DNA has been reported together with their unusual photophysics (Alexandre et al., 2014).

Anyway, non-covalent DNA interactions are a quite complex phenomenon, and usually are characterized by the simultaneous and competitive presence of different interaction modes (Zeglis et al., 2007). Following the seminal work of Barton group (Zeglis et al., 2007), one usually recognizes the presence of minor and major groove-binding, intercalation and insertion. Recently a further mode characterized by the expulsion of a full base-pair from the Watson and Crick pairing (double-insertion) has been reported (Dumont and Monari, 2013).

As well as the interactions, the mechanisms of action of the sensitizers can be quite different, and may follow ground-state or photophysical/photochemical pathways (Dumont and Monari, 2015). The ground-state mechanisms are usually related to the stabilization of peculiar DNA structures or motives and hence to the inhibition of key enzymes or of fundamental biological functions such as duplication. On that aspect particular interest rely on the stabilization of non-canonical DNA structures such as G-quadruplexes (Terenzio et al., 2014). Indeed, the latter are present in telomeres and in gene-regulating regions and hence, their stabilization, and the consequent telomerase inhibition, are related to potential anticancer activity.

On the other hand, excited state sensitization or photosensitization (Epe, 2012) is due to the photoinduced energy or electron transfer happening between the sensitizers and the DNA nucleobases (type I or triplet sensitization), usually involving the population of the triplet state manifold (Victoria et al., 2013). In addition to this direct sensitization, upon light absorption the sensitizer may indirectly activate singlet oxygen (type II sensitization) that will ultimately produce oxidative damages especially on guanine bases (Liang et al., 2013; Nogueira et al., 2015). The latter strategies are particularly common in the so-called dynamic phototherapy.

Even if extremely promising and appealing, the design of therapeutic agents interacting with DNA is a complex task still far from a complete rationalization. Indeed, in many cases the molecules are improperly metabolized, while the interaction with DNA happens to be quite weak, or even the penetration of the sensitizer up to the cell nucleus is hampered by the difficulty to pass the different biological membranes (Zhou et al., 2014). In the case of small organic molecules one can also encounter problems related to the low water solubility or to chemical- and photo-instability. As such many of the most widespread covalent or non-covalent, DNA sensitizers are composed by heavy and rare metal cations, such as Ruthenium (Huang et al., 2014; Véry et al., 2014). This fact leads to a two-fold problem: On the one hand, the high cost and rarity of such metals, precluding a large scale use, and on the other hand the relatively high toxicity of endogenous compounds such as Ruthenium that is known to induce a considerable oxidative stress (Kapitza et al., 2005). Hence the possibility to use less toxic, abundant, and cheap metals, such as iron is of major interest.

The DNA/sensitizers interactions being an extremely complex phenomena, its rationalization calls for a combined effort in which molecular modeling couples with experimental chemistry and biology (Monari et al., 2015). This interdisciplinary approach will allow a multiscale vision of the process that will go from the electronic and atomistic resolution, up to the characterization in cellular lines, through the rational design and synthesis of novel interactors. Indeed, the capacity of molecular modeling to describe the interactions between drugs and DNA, as well as the mechanisms leading to DNA lesions have been evidenced in different scientific works and in a complete recent review (Dumont and Monari, 2015).

**Figure 1:**
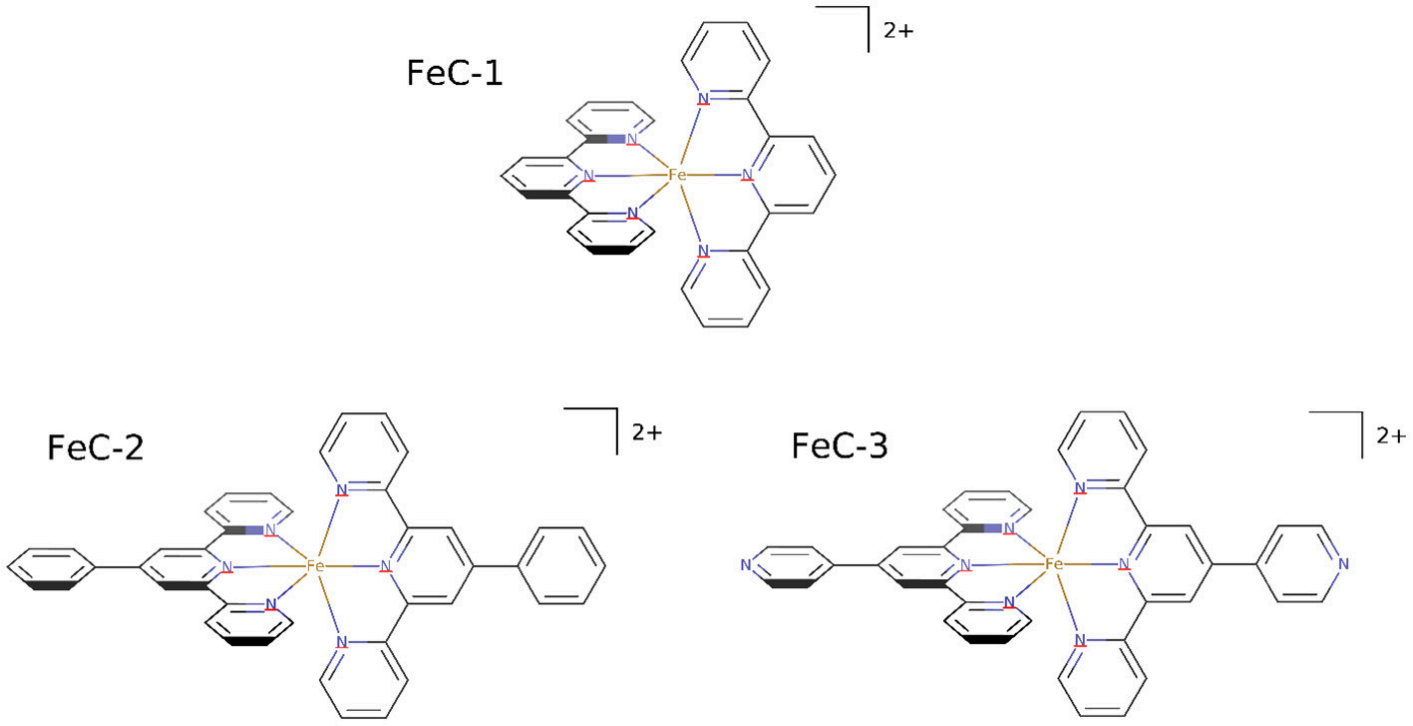
**Molecular formula of the three investigated iron complexes**.

In this work we report the synthesis and characterization of iron complexes bearing different terpyridine ligands (Figure 1). Terpyridine are particularly suitable ligands for iron giving rise to quite stable organometallic complexes, that hence could be thought to resist even in biological environment, moreover the trifold coordination confers a remarkable rigidity to the organometallic complex. These aspects also preclude the possibility to let one coordination site free for interaction with water or reactive oxygen species, hence diminishing the possible toxicity due to oxidative stress. On the other hand, the presence of a large aromatic ligand systems will favor the non-covalent binding with DNA by maximizing the possible π-stacking, while also allowing the passage through the biological membranes, due the hydrophobic environment provided by the ligands. On the other hand the positive charge of the complexes, brought by the iron cation might favor interactions with the strongly negatively charged DNA backbone. The interactions with DNA will be characterized by using molecular dynamics (MD) techniques, and proven by UV-vis absorption spectroscopy. Particular emphasis will be put on the analysis of the structural deformation induced to DNA, as well as to the stabilizing interactions allowing the formation of stable aggregates. Furthermore, to prove the emergence of non-negligible biological effects, the influence of the iron complexes on the cells' viability will be tested.

## Materials and methods

### Molecular modeling

The interaction between the three FeC complexes and DNA was modeled by using MD techniques. To this aim two different B-DNA double strands were built *in silico* using the amber nab utility (Case et al., 2015). Both strands were composed of 14 bp, and were homogeneously constituted of poly(dA)-poly(dT) and poly(dG)-poly(dC), respectively. This choice allows to investigate the possible different affinities for guanine or adenine rich regions, indeed since poly(dA)-poly(dT) strands are much more flexible than their poly(dG)-poly(dC) counterparts, significant structural differences can occur. On the other hand, the use of homogeneous DNA strands allows reducing the complexity of the problems, in particular concerning the possible non-equivalent interaction sites. The DNA double strands were modeled by using the amber99 force field, including the bsc0 corrections (Pérez et al., 2007) that were specifically developed to improve the description of the backbone dihedral degrees of freedom and hence are necessary to account for the long-scale dynamics of DNA double-strands.

To model the iron complexes we parameterized three specific force fields respecting the following protocol: the ligands parameters were obtained by the generalized amber force field (gaff) (Wang et al., 2004), while specific parameters to correctly reproduce the angles and dihedrals around the octahedral coordination sphere of iron were manually imposed to respect the quantum mechanical (QM) obtained equilibrium geometries. Finally point charges were assigned using the Restrained Electrostatical Potential (RESP) fitting procedure (Bayly et al., 1993). Electrostatic potentials around the complexes were obtained by the standard amber protocol, i.e., QM Hartree-Fock calculations performed using 6-31G^*^ as basis set. The equilibrium geometry was obtained by density functional theory (DFT) with B3LYP (Stephens et al., 1994) as exchange correlation functional and 6-31G^*^. All QM calculations were performed using Gaussian 09 (Frisch et al., 2009). The force field was validated performing 100 ns MD in a solvated water box and observing no significant deviations from the equilibrium geometry. Force field bonding parameters and charges are given in Supplementary Information.

**Figure 2:**
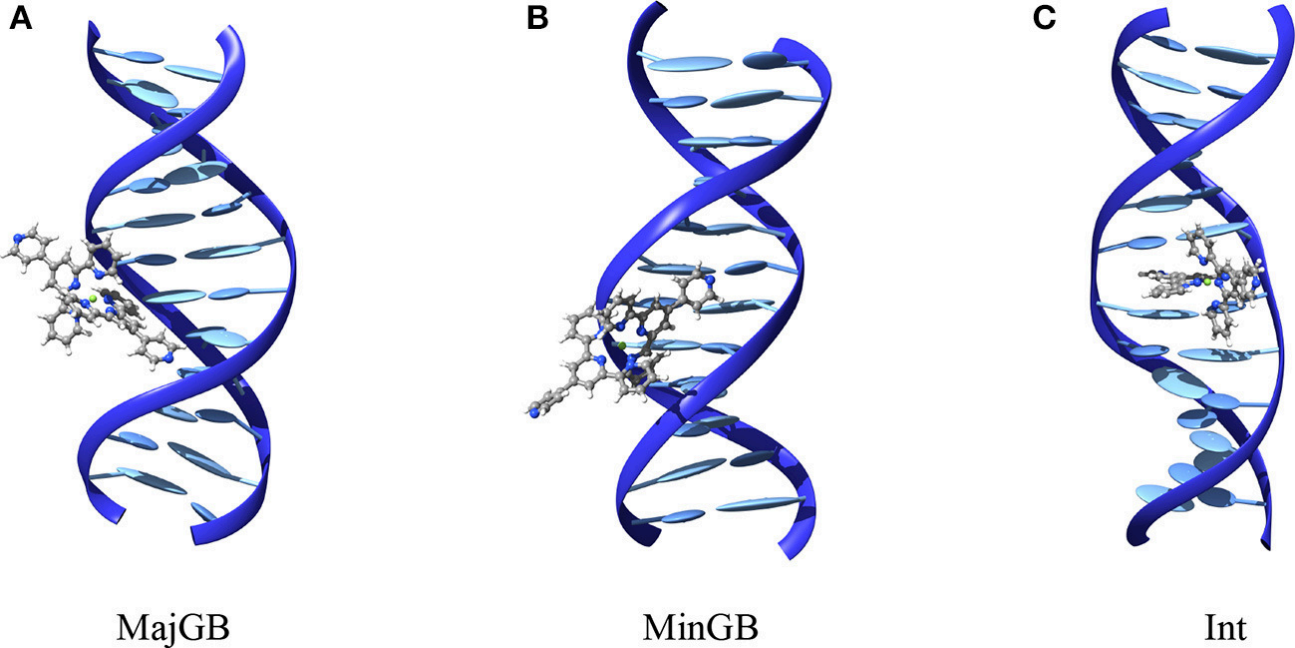
**Structures of the different interaction modes tested extracted from MD simulations**. **(A)** Major groove binding, **(B)** minor groove binding, and **(C)** intercalation. Structures are reported for FeC-3 and poly(dG)-poly(dC) strand.

For both poly(dA)-poly(dT) and poly(dG)-poly(dC) we manually placed each of the three complexes in interaction with the DNA duplex. More specifically (see Figure 2), for each interactor and duplex we built the starting conformation for major groove binding (MajBG), minor groove binding (MinBG), and intercalation (Int), for a total of 18 starting conformations. In all the cases and in order to minimize border effects the metal complex was placed close to the central point of the strand.

Each one of the 18 DNA/FeC complex starting conformation was then solvated in a truncated octahedral water box, with a buffer of 10 Å around DNA, solvent molecules were described by TIP3P force field (Jorgensen et al., 1983). To ensure electroneutrality of the box K^+^ cations have been added to compensate the backbone negative charges. The iron complexes bearing a +2 charge a total of 26 K^+^ has been added to the simulation box. Following a first energy minimization to eliminate bad contacts each system was firstly brought to 300 K via 200 ps MD in the NVT ensemble. Subsequently, a 200 ps equilibration dynamics has been performed in the NPT ensemble to reach a pressure of 1 atm. and the good density. Following the preparation step a 100 ns production runs have been performed in the NPT ensemble at 300 K and 1 atm. for each of the systems. All MD trajectories have been obtained using the amber 2015 suite of codes and its GPU expansion (Case et al., 2015). For all simulations, the periodic unit cell is a truncated octahedron with 70.5 A long edges and ~25,600 water molecules.

MD trajectories have been analyzed to identify the occurrence of stable interactor/drug aggregates. In the case of the occurrence of stable aggregates the DNA strand behavior has been analyzed using the Curves+ software (Lavéry et al., 2009) in order to quantify its distortion due to the interaction with the iron complex. In particular the bending of the DNA axis all along the trajectory is obtained, as well as the local backbone, groove, and base-pair geometrical parameters. Furthermore, the non-covalent interaction taking place between DNA and the different complexes have been evidenced and classified using the Non Covalent Interaction (NCI) methodology (Johnson et al., 2010) based on the analysis of the peaks appearing in the electron density at low intensities and of their curvature (Hessian). Most notably NCI is able to discriminate between steric clashes (i.e., repulsive interactions), dispersion and π-stacking (i.e., attractive interactions with close to zero curvature), and hydrogen-bonding (i.e., attractive interactions with high curvature). NCI analysis has been performed using the NCIPlot code and the promolecular implementation (Contreras-Garcia et al., 2011).

### Synthesis and characterization

**Scheme 1:**
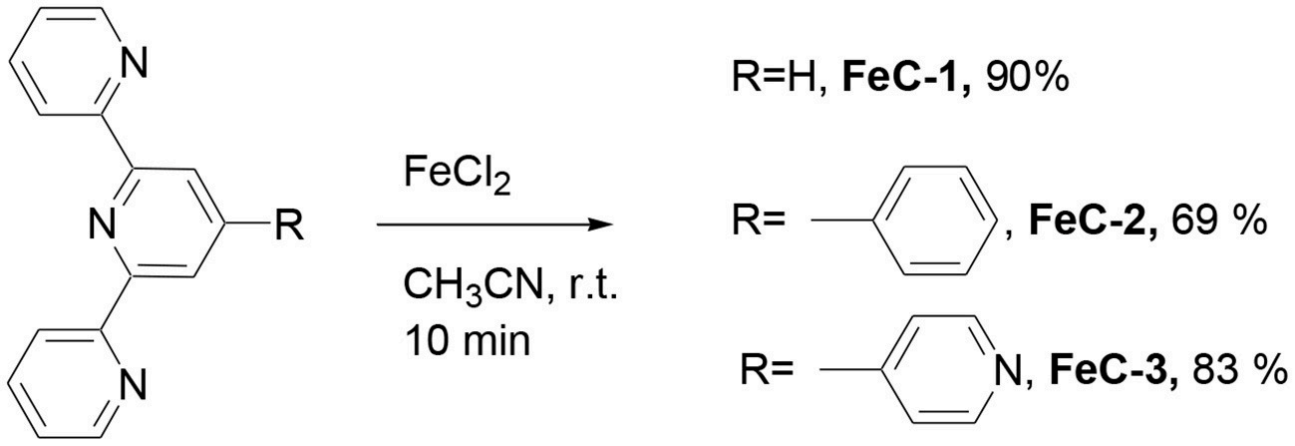
**Synthesis of complexes studied in this work**.

The terpyridine ligands required for the synthesis of complexes have been prepared using the Kröhnke reaction between 2-acetylpyridine and the appropriate aryl aldehyde in the presence of ammonia (Constable and Cargill Thompson, 1992; Moya et al., 2001). The homoleptic complexes **FeC-1**, (Constable and Cargill Thompson, 1992), **FeC-2** (Krass et al., 2001), and **FeC-3** (Constable and Cargill Thompson, 1992) were obtained in 90, 69, and 83% yield respectively by reacting the appropriate terpyridine ligand (2 equiv) with FeCl_2_ in acetonitrile (Scheme 1).

#### General procedure for preparation of complexes

A solution of FeCl_2_ (106 mg, 0.84 mmoles) in methanol (20 mL) was added to a solution of the appropriate terpyridine (1.68 mmoles) in methanol (20 mL). The reaction mixture was stirred for 10 min until a clear dark purple solution was obtained. Diethyl ether (100 mL) was finally added and the dark purple solid was collected by filtration, washed with diethyl ether, and dried under vacuum.

**FeC-1**. Yield: 90%. NMR ^1^H (200 MHz, CD_3_OD): δ = 7.85 (*d*, *J* = 8.1 Hz, 4H), 7.49 (*t*, *J* = 8.2 Hz, 2H), 7.41 (*d*, *J* = 8.2 Hz, 4H), 6.68 (m, 4H), 5.90 (*d*, *J* = 3.8 Hz, 8H) ppm. HRMS (ESI) calcd for C_30_H_22_FeN_6_Cl_2_ m/z = 261.0627 [M – 2 Cl]^2+^. Found: 261.0683.
**FeC-2**. Yield: 69%. NMR ^1^H (200 MHz, DMSO-d6) 9.70 (s, 4H), 9.09 (*d*, *J* = 8.5 Hz, 4H), 8.57 (*d*, *J* = 7.4 Hz, 2H), 8.05 (*t*, *J* = 7.4 Hz, 4H), 7.85 (*t*, *J* = 7.2 Hz, 4H), 7.78 (*t*, *J* = 7.0 Hz, 2H,), 7.3 (*d*, *J* = 5.1 Hz, 4H), 7.2 (*t*, *J* = 6.3 Hz, 4H). HRMS (ESI) calcd for C_42_H_30_FeN_6_Cl_2_ m/z = 337.0936 [M – 2 Cl]^2+^. Found: 337.0902.
**FeC-3**. Yield: 83%. ^1^H NMR (200 MHz, CD_3_CN): δ = 9.27 (s, 2H), 9.08 (*d, J* = 5.8 Hz, 2H), 8.65 (*d*, *J* = 8.1 Hz, 2H), 8.38 (*d*, *J* = 5.8 Hz, 2H), 7.97 (dt, *J* = 6.7 Hz, 1.2 Hz, 2H), 7.22-7.10 (m, 4H) ppm. HRMS (ESI) calcd for C_40_H_28_FeN_8_Cl_2_ m/z = 338.0888 [M – 2 Cl]^2+^. Found: 338.0906.

The UV-vis absorption spectrum was registered for the FeC-3 molecule interacting with increasing concentration of B-DNA. These assays were performed with a Nanodrop system from Thermo Scientific. One microliter of sample (DNA alone, compounds alone or compounds+DNA) is used for these experiments and the absorbance was measured from 190 to 840 nm.

### Cellular biology

Cellular Viability was tested following a rather classical protocol as detailed in the following.

#### Cell culture

The MCF 10 A cell line is a non-tumorigenic human epithelial cell line (ATCC). Cells were grown in DMEM /F12 Ham's Mixture (Invitrogen) supplemented with 5% Equine Serum (Gibco), EGF 20 ng/mL (Sigma), insulin 10 μg/ml (Sigma), hydrocortisone 0.5 μg/mL (Sigma), cholera toxin 100 ng/mL (Sigma), 50 μg/mL of gentamycin (Sigma) at 37°C, 5% CO_2_.

#### Cell viability assay

Cells were seeded at 2 × 10^3^ cell per well in a 96 well plate with 0.1 mL of culture medium and were treated 24 h later with molecule during 48 h. Viability of cells was evaluated using crystal violet staining. Following treatment, the cells were washed with phosphate buffer saline (PBS) solution and fixed with 0.1 mL of paraformaldehyde at 4% for 20 min. After a washing with PBS, cells were stained with 0.1 mL of crystal violet at 0.1% dye for 30 min and washed with H_2_O. The crystals on the plate were then dissolved with 0.1 mL of acetic acid at 10% and absorbance at 595 nm wavelength with a microplate reader (Victor X, Perkin Elmer) was determined.

#### Statistical analysis

All results are represented as mean value ± SEM. Statistical analyses were performed by using Student's *t*-test which compared untreated vs. treated cells. Statistically significant results were represented as follows: ^*^p < 0.05, ^**^p < 0.01, ^***^p < 0.001.

## Results

### Stability of the aggregates with DNA

The behavior of the different FeC complexes with the two DNA strands turns out to be quite complex showing a number of peculiarities that should be taken into accounts. Results are summarized in Table 1.

**Table 1:** **Behavior of the different iron complexes interacting with DNA, as a function of the strand and of the interaction mode**.

		**poly(dA)-poly(dT)**	**poly(dG)-poly(dC)**
**FeC-1**	*MajGB*	Unstable	Unstable
	*MinGB*	Stable (Diffuses in the Groove)	Stable (Diffuses in the Groove)
	*Int*	Unstable	Unstable (Evolves toward MinGB)
**FeC-2**	*MajBG*	Unstable	Unstable
	*MinGB*	Stable	Stable
	*Int*	Stable	Stable
**FeC-3**	*MajGB*	Metastable (10 ns)	Metastable (10 ns)
	*MinGB*	Stable	Stable
	*Int*	Stable	Stable

First of all one can observe a strong difference between the three iron complexes, indeed while FeC-1 is only stable in MinBG binding, FeC-2, and FeC-3, thanks to the presence of an additional phenyl ring have large enough π-conjugated systems allowing the intercalation binding mode to be sufficiently stable. It has also to be noted that MinBG in the case of FeC-1 appears less stable compared to the other two complexes. Indeed, during the 100 ns trajectory, FeC-1 slides along the minor groove, hence exploring different interaction sites of the DNA. Interesting enough, the Int starting structure for FeC-1 in poly(dG)-poly(dC) evolves quite rapidly with the drug being ejected toward the major groove.

**Figure 3:**
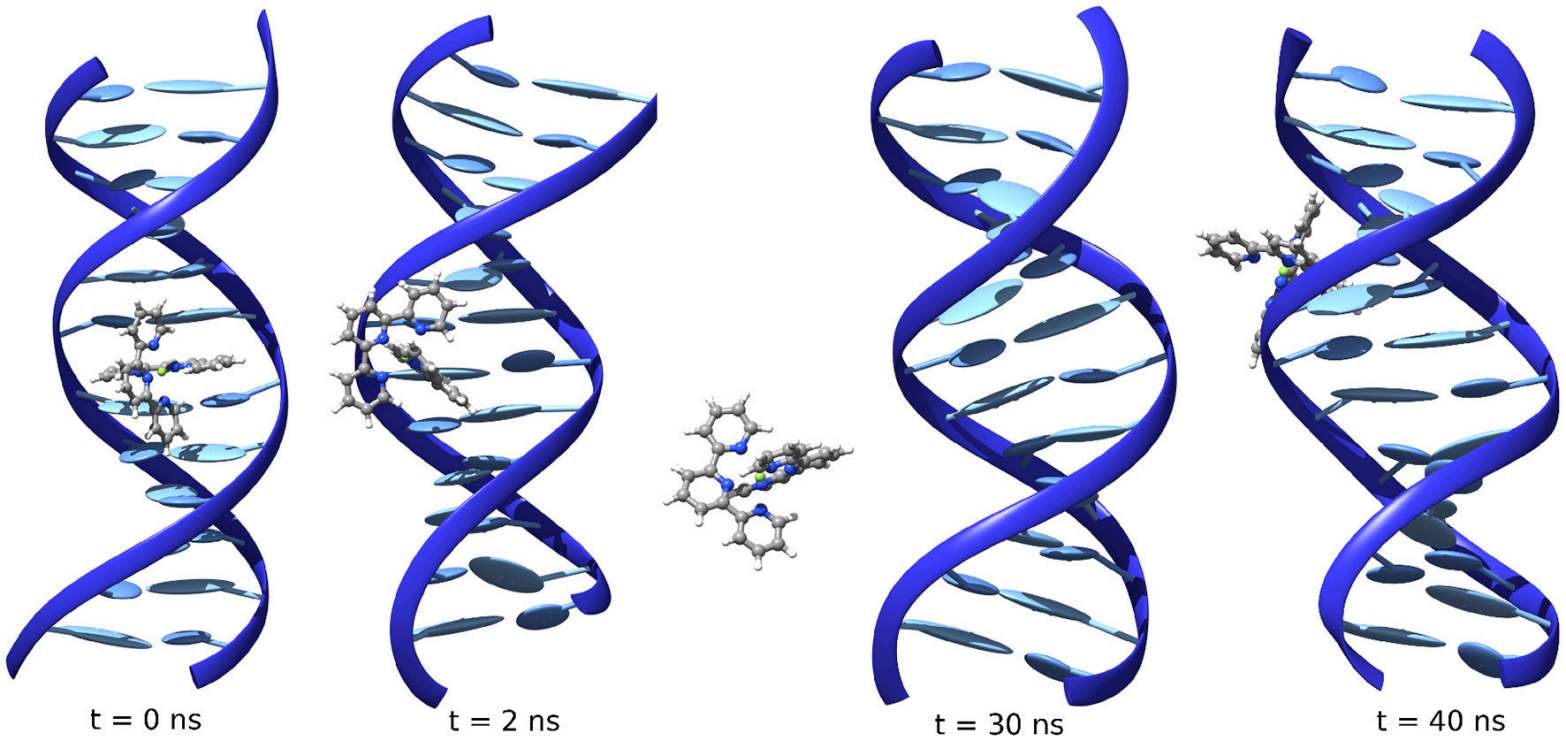
**Evolution of the position of FeC-1 interacting with poly(dG)-poly(dC) starting from intercalation**.

This situation, however, still represents an unstable mode and the interactor rapidly leaves for the bulk. However, probably due to the electrostatic interactions with the negatively charged DNA, it can subsequently reach a stable position in the minor groove, where it stays until the end of the MD (Figure 3). The evolution from an unstable MajGB to MinGB is also evidenced for some of the trajectories of FeC-2 and FeC-3 confirming a strong affinity of the different complexes for DNA as well as a very low, or non-existent kinetic barrier, for the MinBG processes.

FeC-2 and FeC-3 also give strongly stable aggregates both for MinGB and Int. While a complete characterization of the two would require the calculation of the binding free energy, and eventually the barriers potentially leading to kinetic blockages, our results suggest the simultaneous presence of two competitive binding modes. As far as MajGB is concerned almost no stable aggregate is obtained, with only metastable interactions observed in the case of FeC-2 with poly(dA)-poly(dT) and FeC-3 with both strands. This fact is certainly due to the rather large width of the major-groove that significantly hampers the formation of stabilizing interactions.

The reasons for the occurrence of stable Int aggregates are basically due to the possibility for the ligands to slip in between the base pairs and hence develop an extended π-stacking with the DNA hydrophobic core.

**Figure 4:**
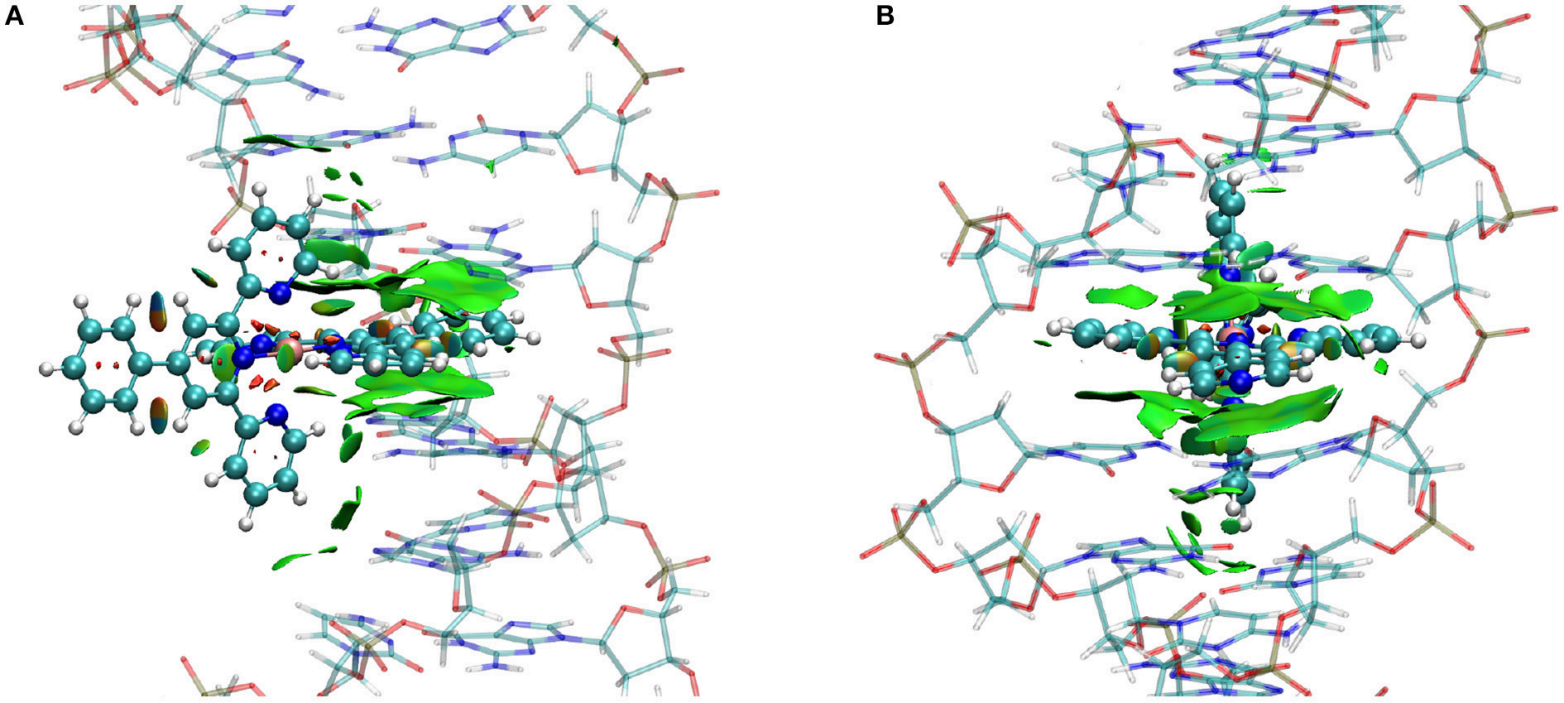
**NCI plot for a representative conformation of FeC-2 (A) and FeC-3 (B) intercalated in the poly(dG)-poly(dC) double strand**. The iron complex is represented in ball and stick representation while the DNA is in shaded licorice. Surfaces represent the non-covalent (π-stacking) interaction.

This is precisely the situation taking place in the case of FeC-2 and FeC-3 as can be observed on the NCI interaction plot reported in Figure 4 for the poly(dG)-poly(dC) double strand. All the others NCI plots are given in Supplementary Information. It is interesting to note that in addition to the dispersion in the intercalation pocket we may observe that the ancillary ligand also develops attractive interactions with the DNA bases, such as dispersion and probably labile hydrogen bonds. This fact implies that the involved bases' position is slightly deviated from the center of the strand, and in particular guanine tends to pivot toward the complex.

**Figure 5:**
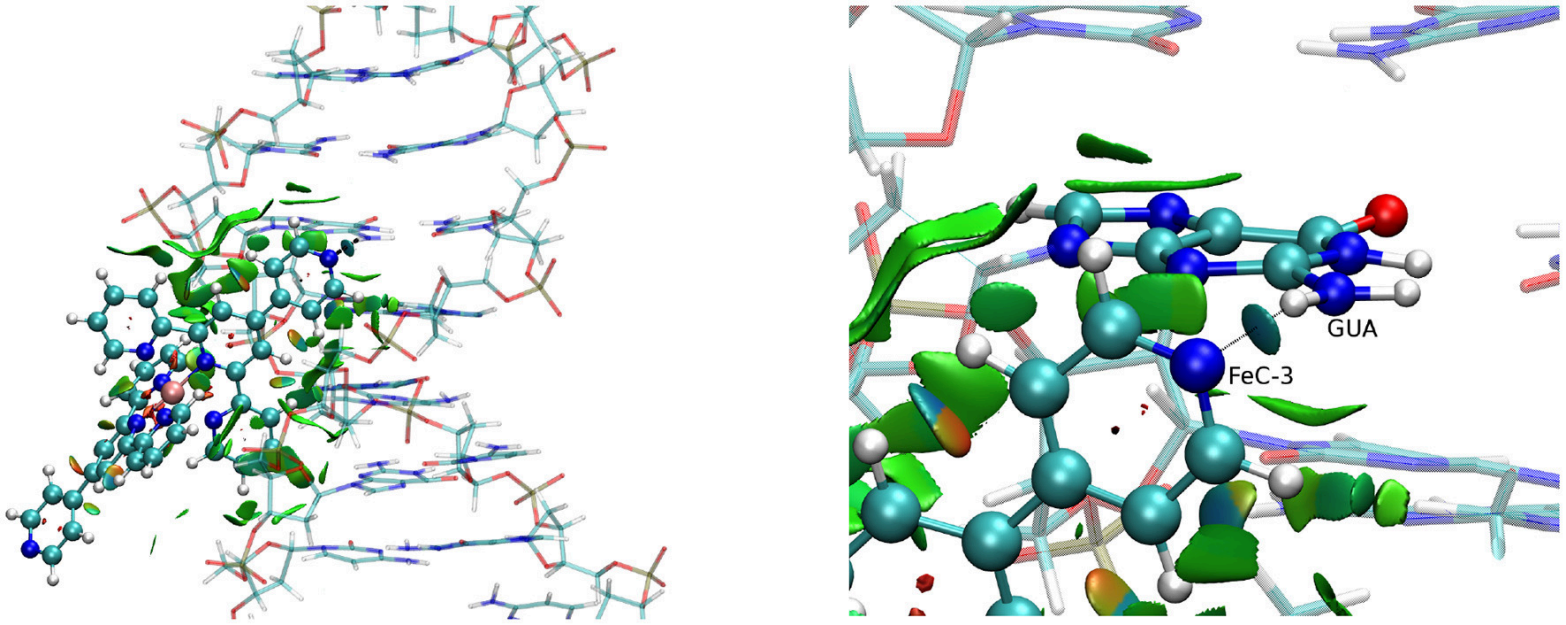
**NCI cartoons for the FeC-3 (poly(dG)-poly(dC)) strand (left) and a zoom showing the interaction with guanine (right)**.

The same important dispersive interaction takes place in the case of the MinGB interaction (Figure 5) with now both the ligands being involved in stabilizing interactions with the DNA backbone and with the basis. Of particular interest is the case of FeC-3. Indeed the nitrogen of the pyridine ligands forms a persistent hydrogen bond with the NH_2_ group of the guanine.

In order to better quantify the different stability we estimated the enthalpy contribution to the binding free energy between the different complexes and DNA by using the Molecular Mechanics-Generalized Born Surface Area (MM-GBSA) methodology (Wang et al., 2006). Although the previous methodology is quite delicate it can nonetheless give a reasonable estimation of the difference affinities. Detailed results are given in Supplementary Information however we can see that while FeC-1 has binding free energies lower than −20 Kcal/mol, FeC-2 and FeC-3 exhibits a much larger stabilization with the MinGB accounting for roughly −25 Kcal/mol and Int for about −38 Kcal/mol. Interestingly, MinGB appears systemically favorable in the case of poly(dA)-poly(dT) while Int is less sensitive to the strand but gives lower MM-GBSA free energies for the FeC-3 ligand.

**Figure 6:**
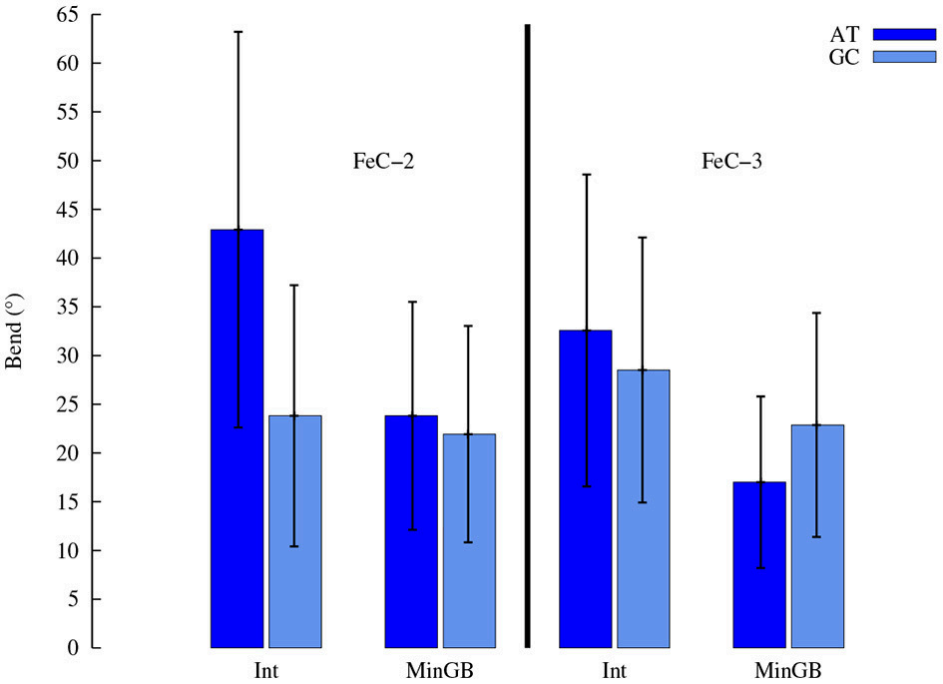
**Average global helix bending induced by FeC-2 and FeC-3**. Error bars represent the standard deviation, the relative high value being due to the relative short length of the double strands.

As far as the structural parameters are concerned a great difference is observed, as expected, between the different binding modes. In the following we will concentrate our analysis onto the most stable interaction modes. One of the most straightforward structural information that can be obtained by MD is the global helical bending. The average value of the bending for the different complexes in the stable interaction modes is reported in Table 2 and Figure 6 and also compared to the average value obtained for the native B-DNA that happens to be close to 20° for both double strands. One can see that while the MinGB mode does not induce a significant distortion, the situation in the case of the Int mode is different. Indeed, especially for the intercalated FeC-2 case, pronounced bending values (~43°) coexist with relative small one (~20°). High bending happens in the cases when the sensitizers is only partially intercalated, such as FeC-2 on the poly(dA)-poly(dT) sequence, the sterical constrains imposed produce a kink and a higher bending of the helix. This occurrence was already observed in crystal structures of Ruthenium complexes interacting with DNA by Barton and coworkers (Zeglis et al., 2007) and by Hébraud's group (Jia et al., 2015), who explicitly spoke of “semi-intercalation.” On the other hand in the case of classical intercalation with the complex slipping deeply inside the DNA hydrophobic core the bending is strongly reduced as for the FeC-2 complex on poly(dG)-poly(dC).

**Table 2:** **Average values of the DNA helix global bending for the different stable aggregates and for free B-DNA**.

		**poly(dA)-poly(dT)**	**poly(dG)-poly(dC)**
**Free B-DNA**		23°	20°
**FeC-1**	*MinGB*	21°	24°
**FeC-2**	*MinGB*	24°	22°
	*Int*	43°	24°
**FeC-3**	*MinGB*	17°	23°
	*Int*	32°	29°

**Figure 7:**
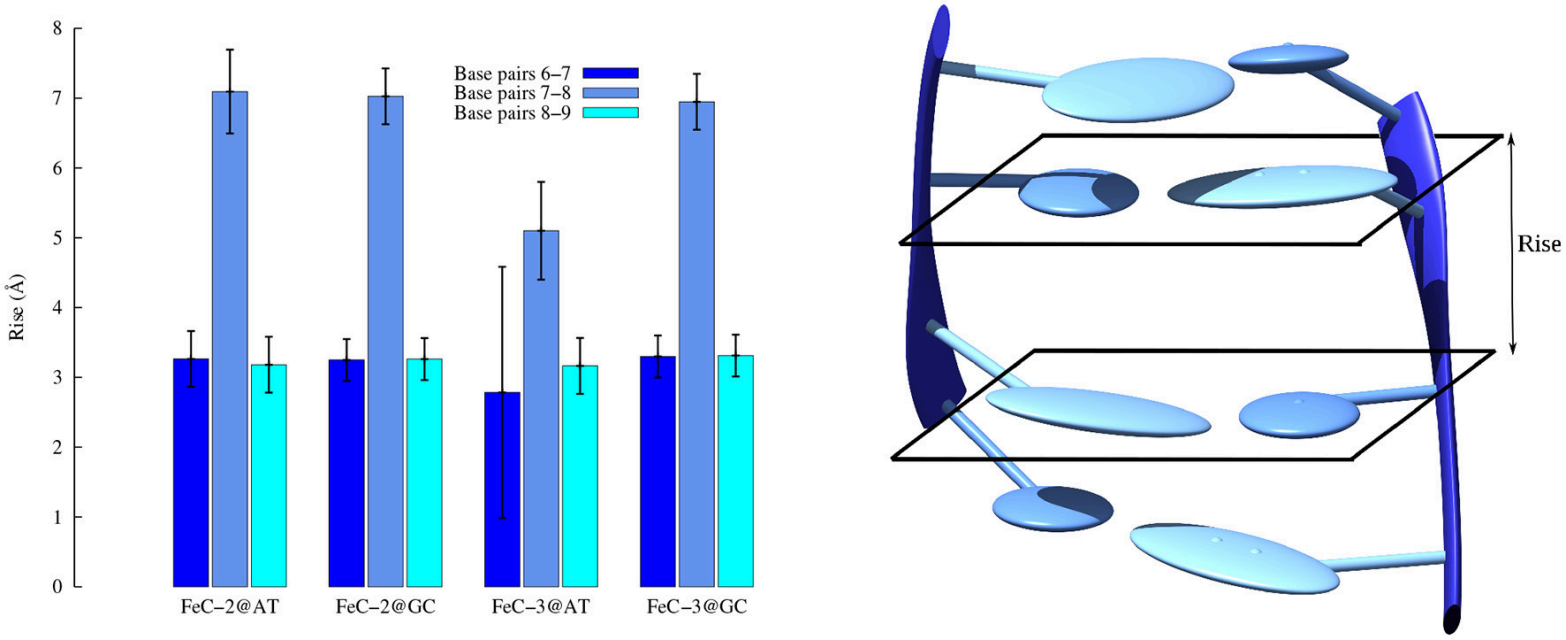
**Average rise parameter for DNA interacting with FeC-2 and FeC-3**. The complex is intercalated in between base pairs 7–8. On the right a cartoon representation of the rise parameter.

As far as the base-pair parameters are concerned, as expected, MinGB produces only slight deviations from the ideal B-DNA structures, while larger deformations are observed for intercalation. In Figure 7 we report the average value of the rise parameter, i.e., the base pairs distance along the DNA axis.

**Figure 8:**
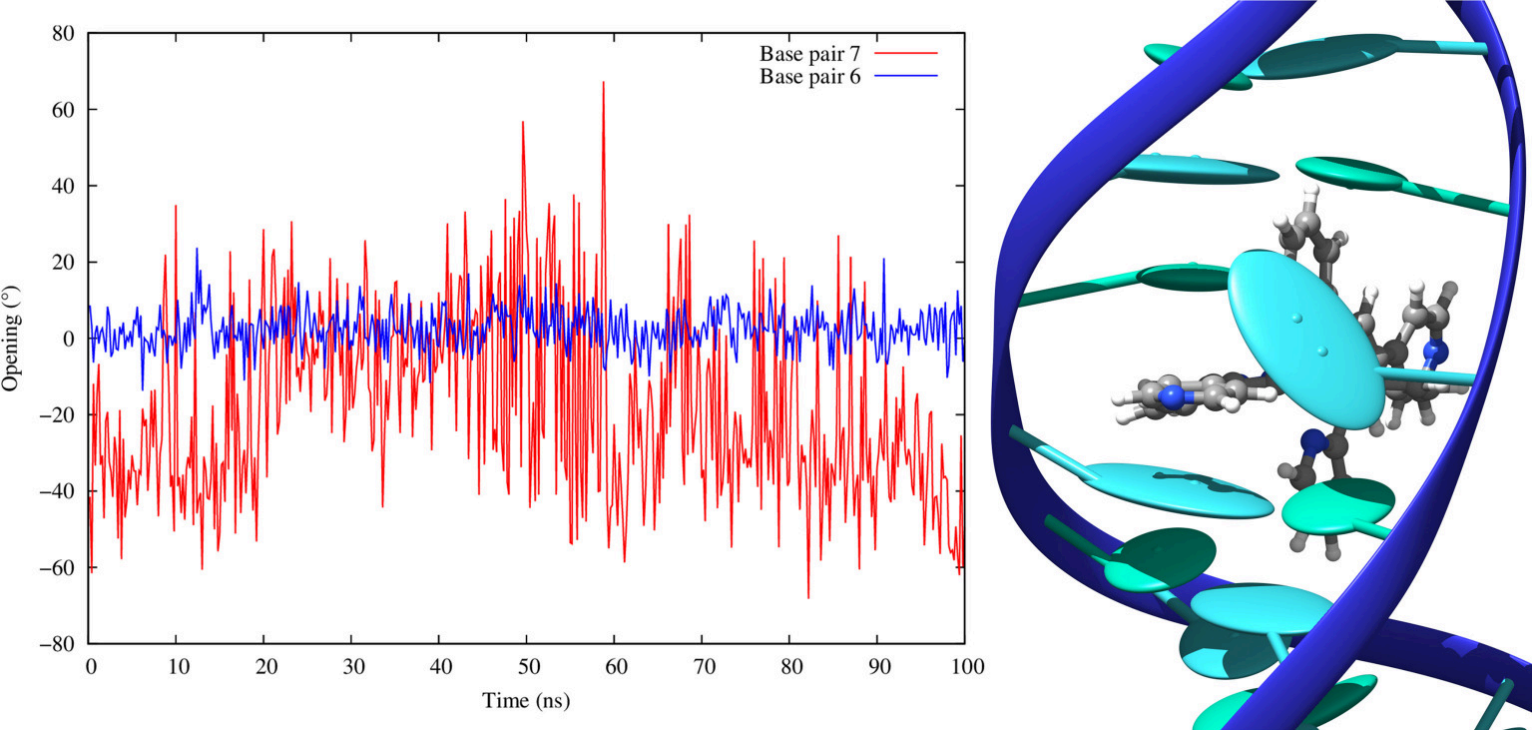
**Time evolution of the opening parameter for base pair 6 and 7 in the case of intercalated FeC-3 in the poly(dG)-poly(dC) double strand**. On the right we report the cartoon of a representative conformation obtained during the MD showing the partial insertion and the ejection of Guanine 7.

As expected the value strongly increases for the couple of base pairs in between which the sensitizers slips, this deformation being necessary to create the intercalation pocket. This value is in most of the cases around 7.00 Å, i.e., almost the double of the ideal 3.4 Å distance, a situation quite common for intercalation (Jia et al., 2015). On the other hand FeC-3 interacting with poly(dG)-poly(dC) has a lower average rise of around 5.2 Å. This occurrence can be explained by the fact that in this case we are facing an intermediate situation between intercalation and insertion, with FeC-3 sensitizer almost kicking one base (namely Guanine 7) out of the Watson and Crick pairing. The partial ejection of Guanine 7 from the Watson and Crick pairing is also responsible for the larger standard deviation of the rise parameter involving this base. This situation is better illustrated in Figure 8 where we report the time series of the opening parameter for GC base pair 7 and the close by GC 6, together with a cartoon representation of a representative snapshot. We remind that opening parameter represents the angle between the base pairs (Lavéry et al., 2009), as such small values indicate a good Watson and Crick pairing, while large values are indicative of a disruption of the pairing and partial ejection of one or both bases. Indeed, while the opening parameter for base pair 6 is close to zero all along the MD trajectory, in the case of base pair 7 and because of the partial ejection of the guanine evidenced in the cartoon, opening experiences oscillations of more than 40°.

**Figure 9:**
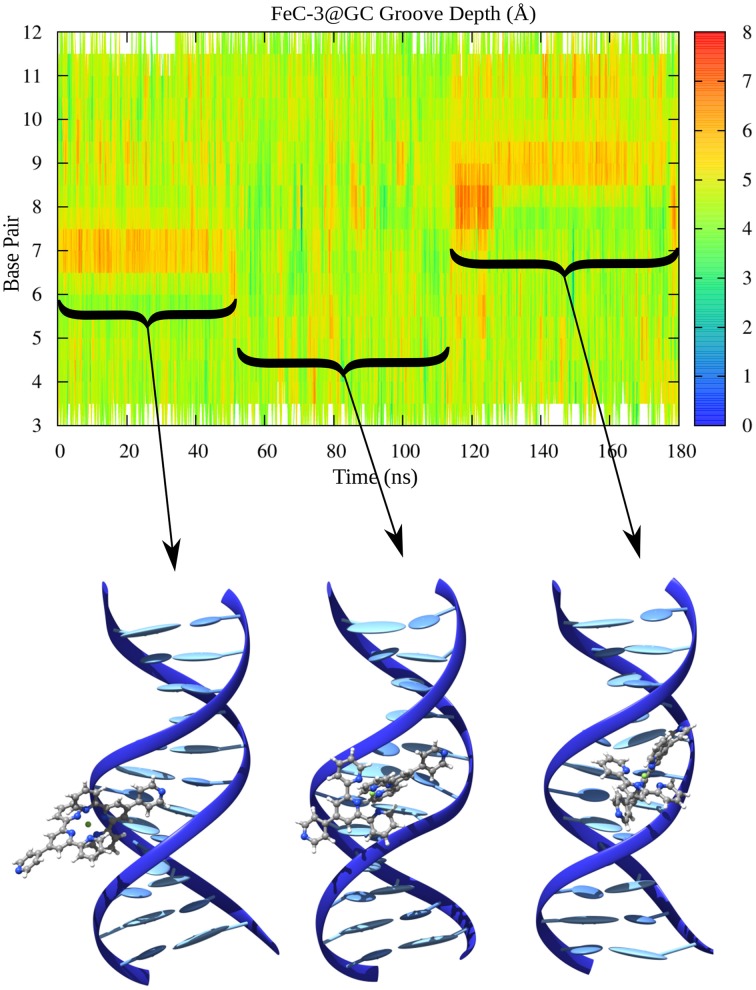
**Minor Groove width for FeC-3 interacting with poly(dG)-poly(dC)**. Representative conformations of the equilibrium modes are also represented in cartoon.

In the case of MinGB, on the other hand, we systematically observe a local enlargement of the groove spanning a region of more or less two or three bases around the sensitizer. This deformation is necessary to accommodate the groove binder minimizing sterical hindrance. Notably the diffusion of the interactor along the groove is strongly coupled with the groove width deformation and is made possible by the enlargement of a wider area of the groove. A particularly illustrative case is the one observed in Figure 9 corresponding to MinGB of FeC-3 in the poly(dG)-poly(dG) strand. In this case we may observe a first stable interaction mode with the sensitizers inducing an augmentation of the width around the 7th base pair. After around 40 ns we observe the sudden change of the groove parameter correlated with the change in orientation of FeC-3 that almost turns of about 180° to stabilize back again in MinGB on the 9th base pair position.

Although different interaction modes and complex situation are observed it is evident that the FeC complex series may strongly interact with DNA and hence may indeed experience a strong biological effect that should be analyzed carefully.

### UV-vis spectroscopy

Iron-II terpyridin compounds are characterized by a well-defined spectrum in the visible and near UV region (Duchanois et al., 2015). In particular, two bands constitute a typical spectroscopical signature of those compounds: a sharp band at around 550 nm due to a metal-to-ligand (MLCT) transition and a band in the UV region (~330 nm) mostly due to intraligand (IL) transitions. The latter bands are relatively intense and happen in a spectral region were DNA is not absorbing, hence facilitating the analysis of the results.

**Figure 10:**
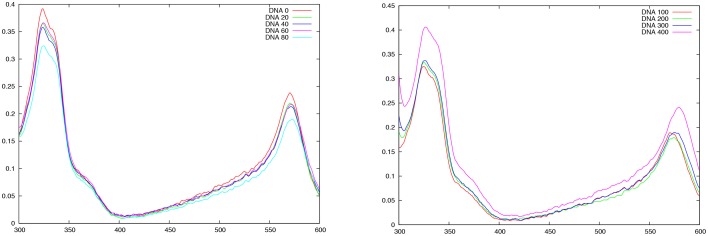
**Absorption spectrum of FeC-3 (100 μM) in presence of increasing DNA concentration**. **Left panel** from 0 to 80 μM B-DNA, **Right panel** from 100 to 400 μM B-DNA

In Figure 10 we report the absorption spectrum recorded for FeC-3 with increasing concentration of B-DNA. Small DNA concentrations (Figure 10 left panel) induce a decrease of the absorption intensities of the two main peaks while the absorption maxima are slightly shifted toward shorter wavelengths. On the other hand, starting from a B-DNA concentration of 100 μM the effect is reversed and now the intensity is enhanced upon increasing DNA concentration. The two different behaviors happening coherently for different concentration ranges can be interpreted as the interplay between two different interaction modes, one dominating at low DNA concentration and the other at higher. Globally, the modification of FeC-3 absorption spectrum in presence of DNA is a clear confirmation that the chromophore is interacting with DNA and hence acting as a sensitizer. However, to clearly unravel and discriminate between the interaction modes one should consider more advanced spectroscopic techniques, such as circular dichroism or even nuclear magnetic resonance.

### Cell viability essays

**Figure 11:**
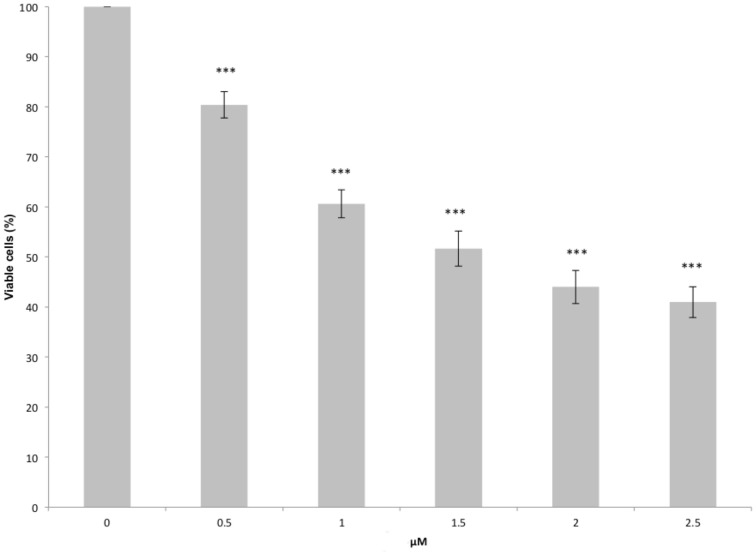
**FeC-3 induced decrease of the viability of MCF10A cells**. Cells were treated with different concentrations of sensitizer for 48 h. Viability was determined by the crystal violet assay. Graphical results represent the mean of 5 independent experiments ± the standard error mean (SEM). Differences between untreated and treated cells were statistically significant for ^***^p < 0.001.

To further prove that our iron complexes are indeed characterized by a non-negligible biological activity we performed *in vitro* viability test on a healthy human cellular lines, following the protocol illustrated in materials and methods. The results are collected in Figure 11, in which we report the effect of the application of an increasing concentration of FeC-3. Although, the other FeC complexes also appear to show biological activity the results are less clear because of solubility problems that could make the interpretation more cumbersome, hence we will limit our prove of concept only to the last molecules of the series, that was also shown by molecular modeling to exhibit the higher affinity toward DNA.

In particular it is evident that even at very low drug concentration a statistically significant decrease of the cell viability is observed, with a reduction of around 20% of the population of the cell line for a dose of 0.5 μM. Increasing the drug concentration induces a significant decrease in viability, with the reduction of 50% of the cell line around 1.5 and 2.0 μM. The observed dose-dependent toxicity is quite promising since it is correlated to a specific mode of action. Although a larger range of concentration would be needed to draw general conclusions our data can be reasonably fitted by an exponential law:

(1) $$\frac{n}{n_{0}}=e^{-\frac{c}{\Gamma }}$$

where *n/n*_0_ is the cell viability, *c* the sensitizer concentration and Γ the characteristic concentration. By fitting the data reported in Figure 10 via the Equation (1) we obtain a characteristic concentration Γ = 2.40 ±0.12 μ*M*. Applying this model we obtain a 50% viability concentration, corresponding to the IC_50_, of 1.66 μM.

Although, the viability cell alone is clearly not sufficient to unambiguously prove the presence of a DNA sensitization, it clearly underlines an inherent biologic and toxic effect of those iron complexes. Furthermore, our complex strongly resembles Ruthenium complexes, whose interaction with DNA has been proven by fluorescence studies (Huang et al., 2014), however the substitution of ruthenium by the more biological compatible iron could represent a significant step forward. We would also like to point out that, even if iron II is known to induce a strong oxidative stress, in particular via the production of reactive oxygen species via the Fenton reaction, the former pathway is unlikely to happen in our case. Indeed, in the FeC series the metal center is shielded by the ligand, and most notably no labile or vacant coordination sites are present to initiate the H_2_O_2_ activation. Furthermore, the hydrophobic ligands also provide shielding to the two formal positive charges of the metal cation, i.e., allowing for the cytoplasmatic, and most probably nuclear membrane permeation.

## Discussion and conclusion

We have reported the study of the interactions taking place between three iron-terpyridine complexes and the B-DNA. By using MD we have unequivocally proved the existence of different stable interaction modes. In particular while MinBG happens to be a stable mode for all the three complexes, intercalation is only possible in the case of sufficiently large enough π-conjugated ligands. MajBG only give raise to metastable aggregates. However, it was also shown that the metastable modes might easily evolve toward more stable ones, such as going from MajGB to MinGB. This fact on the one side proves that the iron-complex interacts strongly enough with the duplex, and on the other side that no significant kinetic barrier exist for the groove-binding modes.

The non-covalent interactions developing between the iron complexes and the DNA duplex have been analyzed and it has been evidenced that dispersion interactions are dominant in the case of Int while electrostatic attraction from the negatively charged backbone are the main responsible for the MinGB. Indeed the lack of a sufficiently extended non-covalent interaction network for MajGB explains the instability of this mode. Interestingly enough, in the case of FeC-3, the appearance of a hydrogen bond between the interactor and guanine has been evidenced in the MinGB. On the other hand the DNA deformation due to the interaction appears quite small and generally local, such as the formation of a large enough intercalation pocket. This facts also point toward favorable interaction. The presence of stable interactions has also been confirmed by MM-GBSA methodology that allows estimating a favorable binding free-energy, also pinpointing a larger stabilization free-energy in the case of intercalation mode.

Experimentally the formation of stable DNA-interactor adducts has been confirmed thanks to UV/V is spectroscopy. Indeed, we have evidenced a DNA concentration dependent modification of the typical spectroscopic signature of iron terpyridine complexes. Furthermore, two specific regimes are evidenced, one dominating at low DNA concentrations, while the second one is evident at larger DNA concentration. This spectroscopic evidence seem to highlight the existence of two competitive binding modes, however more advanced spectroscopies should be necessary to unravel all the details of the interactions.

The biological activity of the aforementioned complexes has also been proved by cellular biology. In particular it has been evidenced that all the three complexes have a toxic effect on healthy cellular lines. Dose dependence has been evidenced as well as a differential effect between the three complexes. Although iron II could also deploy toxic effects by inducing oxidative stress it has to be noted that the iron is strongly protected by the ligand, for instance no vacant coordination site exist that could induce Fenton reaction. Cell viability assays alone cannot provide a full resolution of all the possible interplay between interactions with biological macromolecules and resulting toxicity. However, as confirmed by molecular modeling and by UV-Vis spectroscopy interaction of FeC complexes with DNA duplexes is evident. Furthermore, this interaction taking place especially with DNA in B-form we may speculate it will be mostly favored during the duplication cycle of the cell. Relatively, stable interactions with external drugs can stabilize the double helical structure of DNA hence inhibiting the duplication preventing the opening of the Watson-and-Crick coupling. This factor, as well as induced structural modifications preventing recognition by and interaction with specific enzymes can certainly be at the origin of the toxicity of the drug with a rather common mechanism for cytotoxic drugs (Florea and Büsselberg, 2011). On the other hand due to the extremely short life-time of excited states of similar iron complexes (Duchanois et al., 2015) and in particular due to their ultrafast deactivation through metal centered triplet states, it seems highly improbable to hypothesize phototoxicity resulting from DNA photosensitization either via energy or electron transfer or via singlet oxygen activation. This aspect is certainly in contrast with the mechanism of action of other sensitizers, such as Ruthenium complexes or porphyrines (Dumont and Monari, 2015).

In the following we plan to deeply investigate, by using molecular modeling as well as chemical and biological experiences, all the possible reaction pathways induced by the complexes on the DNA, and hence elucidate the induced damages. On the other hand the presence of the different complexes in the nuclei and the cytoplasm will also be quantified and characterized, as well as its effect on different cellular lines.

## Author contributions

HG performed the molecular modeling part, under the supervision of AM. TD performed the synthesis under the supervision of PG. VB performed the cell viability test under the supervision of SG. VB and CB performed the UV-Vis measurements. AM supervised and wrote the article with the participation of all the authors, XA and PB participated in the discussion on the results and in the general planning of the experiment.

### Conflict of interest statement

The authors declare that the research was conducted in the absence of any commercial or financial relationships that could be construed as a potential conflict of interest.

## References

[B1] Alexandre J. Kirsch-De Mesmaeker A. Elias B. (2014). Selective DNA purine base photooxidation by bis-terdentate iridium(iii) polypyridyl and cyclometalated complexes. Inorg. Chem. 53, 1507–1512. 10.1021/ic402476b 24446771

[B2] Alketa T. Karaflou Z. Kljun J. Turel I. Psomas G. Papadopoulos A. N.. (2013). Antioxidant capacity and DNA-interaction studies of zinc complexes with a non- steroidal anti-inflammatory drug, mefenamic acid. J. Inorg. Biochem. 128, 85–96. 10.1016/j.jinorgbio.2013.07.013 23948577

[B3] Bayly C. I. Cieplak P. Cornell W. Kollmann P. A. (1993). A well-behaved electrostatic potential based method using charge restraints for deriving atomic charges–the resp model. J. Phys. Chem. 97, 10269–10280. 10.1021/j100142a004

[B4] Case D. A. Berryman J. T. Betz R. M. Cerutti D. S. Cheatham T. E. Darden T. A. (2015). AMBER 2015. San Francisco, CA: University of California.

[B5] Chantzis A. Very T. Despax S. Issenhuth J. T. Boeglin A. Hébraud P.. (2014). UV-vis absorption spectrum of a novel Ru(II) complex intercalated in DNA: [Ru(2,2′-bipy)(dppz)(2,2′-arpy)]^+^. J. Mol. Model. 20, 2082. 10.1007/s00894-014-2082-2 24562852

[B6] Constable E. C. Cargill Thompson A. M. W. (1992). Ligand reactivity in iron(II) complexes of 4′-(4″-pyridyl)-2-2′:6′-2″-terpyridine. J. Chem. Soc. Dalton Trans. 2947–2950. 10.1039/dt9920002947

[B7] Contreras-Garcia J. Johnson E. R. Chaudret R. Piquemal J.-P. Beratan D. N. Wang W. (2011). NCIPLOT a program for plotting non covalent interactions. J. Chem. Theory Comput. 7, 625–632. 10.1021/ct100641a 21516178 PMC3080048

[B8] Darren G. Morgan M. P. Marmion C. J. (2009). A novel anti-cancer bifunctional platinum drug candidate with dual DNA binding and histone deacetylase inhibitory activity. Chem. Commun. 44, 6735. 10.1039/b916715c 19885462

[B9] Duchanois T. Etienne T. Cebrian C. Liu L. Monari A. Beley M. (2015). An iron-based photosensitizer with extended excited state lifetime: photophysical and photovoltaic properties. Eur. J. Inorg. Chem. 2015, 2469–2477. 10.1002/ejic.201500142

[B10] Dumont E. Monari A. (2013). Benzophenone and DNA: evidence for a double insertion mode and its spectral signature. J. Phys. Chem. Lett. 4, 4119–4124. 10.1021/jz4021475

[B11] Dumont E. Monari A. (2015). Understanding DNA under oxidative stress and sensitization: the role of molecular modeling. Front. Chem. 3:43. 10.3389/fchem.2015.00043 26236706 PMC4500984

[B12] Epe B. (2012). DNA damage spectra induced by photosensitization. Photochem. Photobiol. Sci. 11, 98–106. 10.1039/C1PP05190C 21901212

[B13] Erkkila K. E. Odom T. D. Barton J. K. (1999). Recognition and reaction of metallointercalators with DNA. Chem. Rev. 99, 2777–2795. 10.1021/cr9804341 11749500

[B14] Florea A.-M. Büsselberg D. (2011). Cisplatin as an anti-tumor drug: cellular mechanisms of activity, drug resistance and induced side effects. Cancers 3, 1351–1371. 10.3390/cancers3011351 24212665 PMC3756417

[B15] Frisch M. J. Trucks G. W. Schlegel H. B. Scuseria G. E. Robb M. A. Cheeseman J. R. (2009). Gaussian 09, Revision B.01. Wallingford, CT: Gaussian Inc.

[B16] Huang H. Zhang P. Yu B. Chen Y. Wang J. Ji L. (2014). Targeting nuclear DNA with a cyclometalated dypiridophenazineruthenium(II) complex. J. Med. Chem. 57, 8971–8983. 10.1021/jm501095r 25313823

[B17] Jia F. Despax S. Münch J.-P. Hébraud P. (2015). Flexibility of short ds-DNA intercalated by a dipyridophenazine ligand. Front. Chem. 3:25. 10.3389/fchem.2015.00025 25932461 PMC4399336

[B18] Johnson E. R. Keinan S. Mori-Sánchez P. Contreras-García J. Cohen J. A. Yang W. (2010). Revealing Noncovalent Interactions. J. Am. Chem. Soc. 132, 6498–6506. 10.1021/ja100936w 20394428 PMC2864795

[B19] Jorgensen W. L. Chandrasekhar J. Madura J. D. Impey R. W. Klein M. L. (1983). Comparison of simple potential functions for simulating liquid water. J. Chem. Phys. 79, 926–935. 10.1063/1.445869

[B20] Kapitza S. Jacupeck M. A. Uhl M. Keppler B. K. Marian B. (2005). The heteorcyclic ruthenium(II) complex KP109 (FFC14A) causes DNA damage and oxidative stress in colorectal tumor cells. Cancer Lett. 26, 115–121. 10.1016/j.canlet.2005.01.002 16039951

[B21] Kellet A. O'Connor M. McCann M. McNamara M. Lynch P. Rosair G.. (2011). Bis phenanthroline copper(ii) phthalate complexes are potent *in vitro* antitumour agents with ‘self-activating’ metallo-nuclease and DNA binding properties. Dalton Trans. 40, 1024–1027. 10.1039/c0dt01607a 21165464

[B22] Krass H. Plummer E. A. Haider J. M. Barker P. R. Alcock N. M. Pikramenou Z.. (2001). Immobilization of π-assembled metallo-supramolecular arrays in thin films: from crystal-engineered structures to processable materials. Angew. Chem. Int. Ed. 40, 3862–3865. 10.1002/1521-3773(20011015)40:20<3862::AID-ANIE3862>3.0.CO;2-N 29712124

[B23] Larragy R. Fitzgerald J. Prisecaru A. McKee V. Leonard P. Kellet A. (2015). Protein engineering with artificial chemical nucleases. Chem. Commun. 51, 12908–12911. 10.1039/C5CC04615G 26154944

[B24] Lavéry R. Moakher M. Maddocks J. H. Petkeviciute D. Zakrzewska K. (2009). Conformational analysis of nucleic acids revisited: curves+. Nucleic Acids Res. 37, 5917–5929. 10.1093/nar/gkp608 19625494 PMC2761274

[B25] Liang C. Ye W. Prestwich E. G. Wishnok J. S. Taghizadeh K. Dedon P. C.. (2013). Comparative analysis of four oxidized guanine lesions from reactions of DNA with peroxynitrite, singlet oxygen, and γ-radiation. Chem. Res. Toxicol. 26, 195–202. 10.1021/tx300294d 23140136 PMC3578445

[B26] Molphy Z. Slator C. Chatgilialoglu C. Kellet A. (2015). DNA oxidation profiles of copper phenanthrene chemical nuclease. Front. Chem. 3:28. 10.3389/fchem.2015.00028 25954741 PMC4404973

[B27] Monari A. Dumont E. Chatgilialoglu C. (2015). Editorial: radiation-induced and oxidative DNA damages. Front. Chem. 3:54. 10.3389/fchem.2015.00054 26380254 PMC4548249

[B28] Moya S. A. Pastene R. Bozec R. L. Baricelli P. J. Pardey A. J. Gimeno J. (2001). Metallic carbonyl complexes containing heterocycles nitrogen ligands - Part VI. Re(I), Mn(I), Mo(0), and W(0) compounds with 4′-phenyl-2, 2′:6′,2″-terpyridine. Inorg. Chim. Acta 312, 7–14. 10.1016/S0020-1693(00)00292-9

[B29] Nahid S. Moghadam N. H. (2013). Study on the interaction of the antiviral drug, zidovudine with DNA using neutral red (NR) and methylene blue (MB) dyes. J. Lumin. 134, 629–634. 10.1016/j.jlumin.2012.07.017

[B30] Nelson S. M. Fergusson L. R. Denny W. A. (2007). Non covalent/DNA interactions: minor groove binding agents. Mut. Res. 623, 24–40. 10.1016/j.mrfmmm.2007.03.012 17507044

[B31] Nogueira J. J. Oppel M. González L. (2015). Enhancing intersystem crossing in phenotiazinium dyes by intercalation into DNA. Angew. Chem. Int. Ed. Engl. 54, 4375–4378. 10.1002/anie.201411456 25663283

[B32] Pérez A. Marchán I. Svozil D. Sponer J. Cheatham T. E. Laughto- nand C. A.. (2007). Refinement of the AMBER force field for nucleic acids: improving the description of αγ conformers. Biophys. J. 92, 3817–3829. 10.1529/biophysj.106.097782 17351000 PMC1868997

[B33] Rajendiran V. Murali M. Suresh E. Palaniandavar M. Periasamy V. S. Akbarsha M. A. (2008). Non-covalent DNA binding and cytotoxicity of certain mixed-ligand ruthenium(ii) complexes of 2,2'- dipyridylamine and diimines. Dalton Trans. 16, 2157–2170. 10.1039/b715077f 18398542

[B34] Reedijk J. (2009). Platinum anticancer coordination compounds: study of DNA binding inspires new drug design. Eur. J. Inorg. Chem. 10, 1303–1312. 10.1002/ejic.200900054

[B35] Rescifina A. Zagni C. Varrica M. G. Pistarà V. Corsaro A. (2014). Recent advances in small organic molecules as DNA intercalating agents: Synthesis, activity, and modeling. Eur. J. Med. Chem. 74, 95–115. 10.1016/j.ejmech.2013.11.029 24448420

[B36] Rosenberg B. VanCamp L. (1970). The successful regression of large solid sarcoma 180 tumors by platinum compounds. Cancer Res. 30, 1799–1802. 5457941

[B37] Stephens P. J. Devlin F. J. Chabalowski C. F. Frisch M. J. (1994). Ab initio calculation of vibrational absorption and circular dichroism spectra using density functional force fields. J. Phys. Chem. 98, 11623–11627. 10.1021/j100096a001

[B38] Terenzio A. Bonsignore R. Spinello A. Gentile C. Martorana A. Ducani C. (2014). Selective G-quadruplex stabilizers: schiff-base metal complexes with anticancer activity. RSC Adv. 4, 33245 10.1039/C4RA05355A

[B39] Very T. Ambrosek D. Otsuka M. Gourlaouen C. Assfeld X. Monari A.. (2014). Photophysical properties of of ruthenium (II) polypyridyl DNA intercalators: effects of the molecular surroundings investigated by theory. Chem. Eur. J. 20, 12901–12909. 10.1002/chem.201402963 25145959

[B40] Very T. Despax S. Hébraud P. Monari A. Assfeld X. (2012). Spectral properties of polypyridyl ruthenium complexes intercalated in DNA: insights nto the surroundings effects of Ru[(dppz)(bpy)_2_]^+^. Phys. Chem. Chem. Phys. 14, 12496–12504. 10.1039/c2cp40935f 22700035

[B41] Victoria V. Rodríguez-Muñiz G. M. Cuquerella M. C. Lhiaubet-Vallet V. Miranda M. A. (2013). Photosensitization of DNA by 5-methyl-2-pyrimidone deoxyribonucleoside: (6 - 4) photoproduct as a possible trojan horse. Angew. Chem. Int. Ed. Engl. 52, 6476–6479. 10.1002/anie.201302176 23657994

[B42] Wang J. M. Hu T. J. Xu X. J. (2006). Recent advances in free energy calculations with a combination of molecular mechanics and continuum models. Curr. Comput. Aided Drug Des. 2, 287–306. 10.2174/157340906778226454

[B43] Wang J. Wolf R. M. Caldwell J. W. Kollman P. A. Case D. A. (2004). Development and testing of a general amber force field. J. Comput. Chem. 25, 1157–1174. 10.1002/jcc.20035 15116359

[B44] Zeglis B. M. Pierre V. C. Barton J. K. (2007). Metallo-intercalators and metallo-insertors. Chem. Commun. 4565–4579. 10.1039/b710949k 17989802 PMC2790054

[B45] Zhou J. Chang A. Wang L. Liu Y. Liu X. Shangguan D. (2014). Effects of side chains on DNA binding, cell permeability, nuclear localization and cytotoxicity of 4-aminonaphtalimides. Org. Biomol. Chem. 12, 9207–9215. 10.1039/c4ob01274g 25299620

