# Supplementary material for: Interaction of Iron II Complexes with B-DNA. Insights from Molecular Modeling, Spectroscopy, and Cellular Biology
Source: Front Chem. 2015 Dec 18;3:67. doi: 10.3389/fchem.2015.00067 (PMC4683171; doi:10.3389/fchem.2015.00067)
Supplement: Supplementary file 1 [file DataSheet1.PDF]

Supplementary Informations for

Interaction of Iron II Complexes with B-DNA. Insights from Molecular Modeling and Cellular Biology.

Hugo Gattuso[1], Thibaut Duchanois[2], Vanessa Besancenot[3], Claire Barbieux[3], Xavier Assfeld[1], Philippe Becuwe[3], Philippe C. Gros[2], Stephanie Grandemange[3], Antonio Monari[1*]

[1]Université de Lorraine – Nancy and CNRS, Theory-Simulation-Modeling, SRSMC, Vandoeuvre-lès-Nancy, France
[2]Université de Lorraine – Nancy and CNRS, Hécrin, SRSMC, Vandoeuvre-lès-Nancy, France
[3]Université de Lorraine – Nancy and CNRS, CRAN, Vandoeuvre-lès-Nancy, France

I) NCI plots for representative DNA/FeC interaction cases.

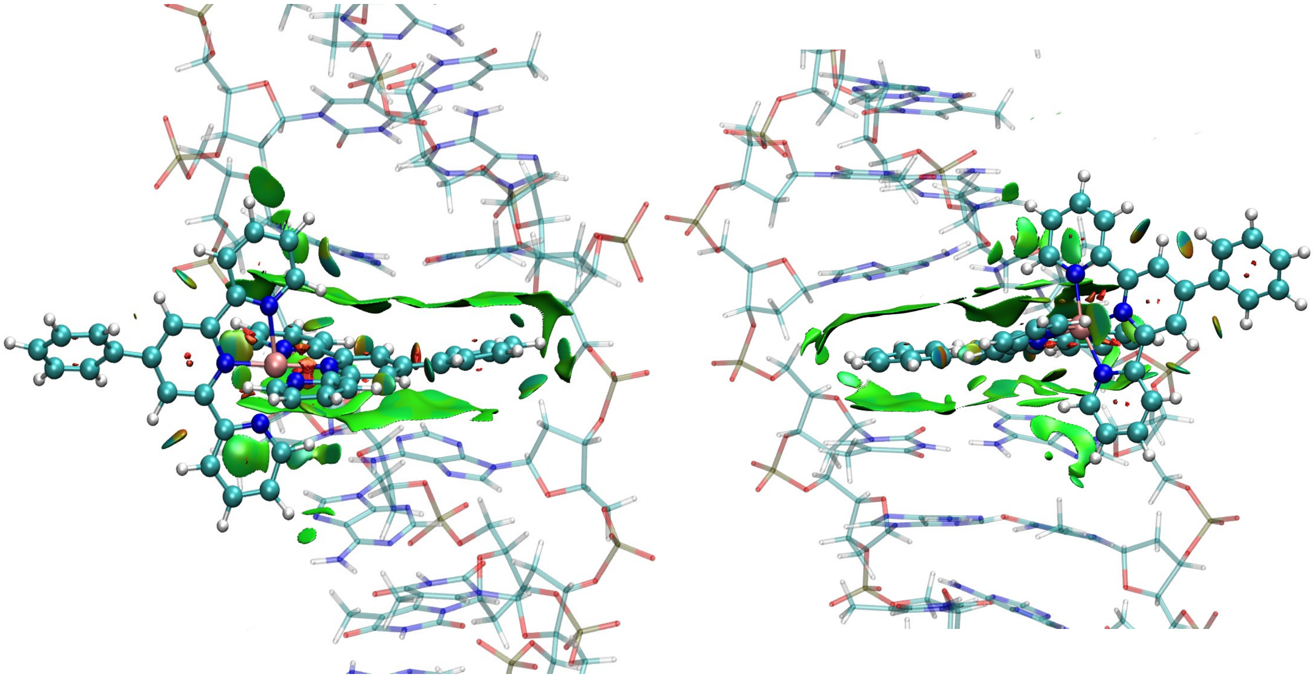

Figure 1: NCI plots for the two main intercalation binding conformations between poly(dA)-poly(dT) and FeC-2.

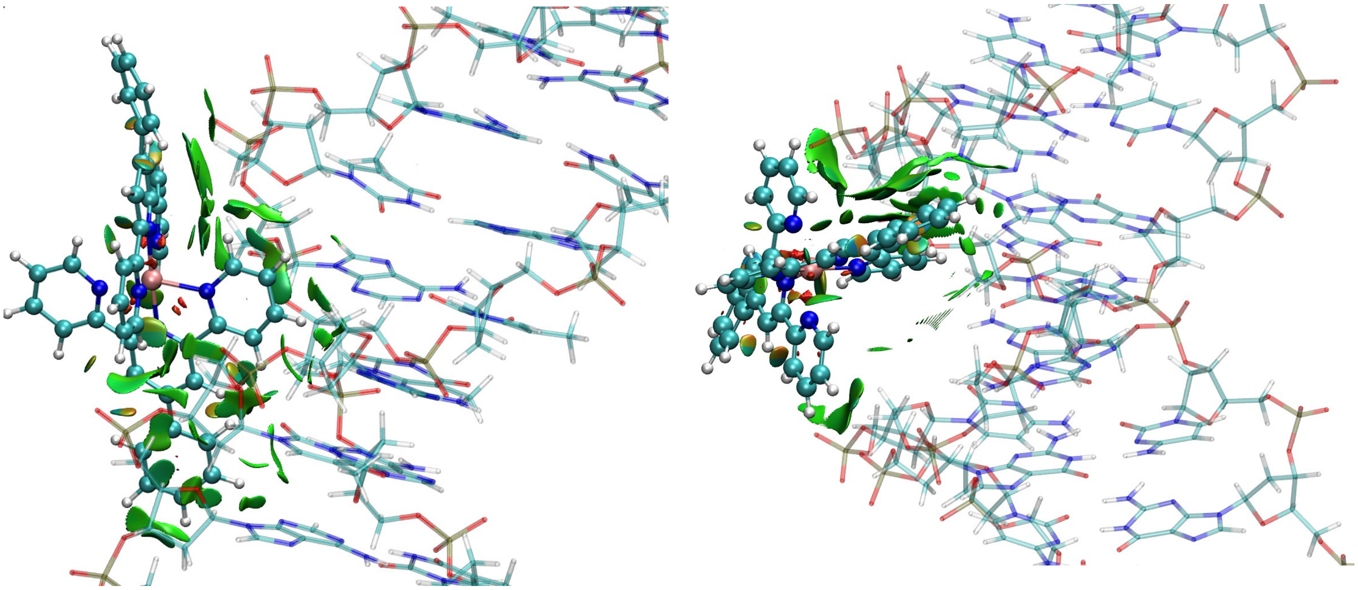

Figure 2: NCI plots for the minor-groove binding of FeC-2 in poly(dA)-poly(dT) (left) and poly(dG)-poly(dC).

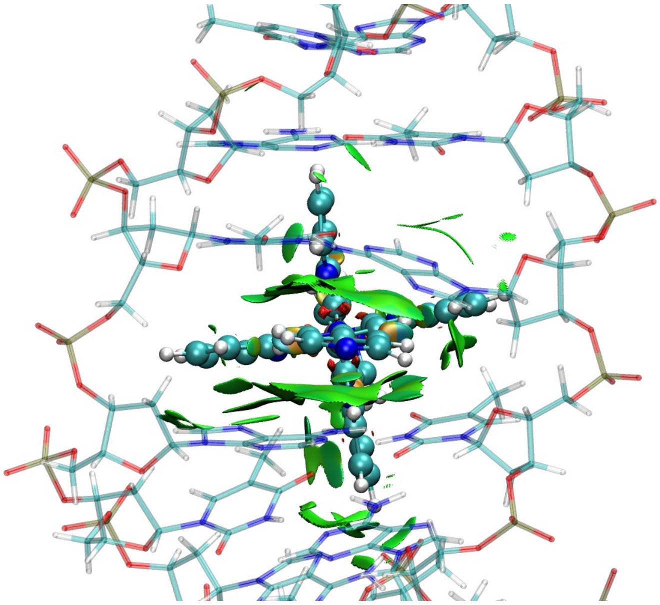

Figure 3: NCI plot for the intercalation of FeC-3 in poly(dA)-poly(dT)

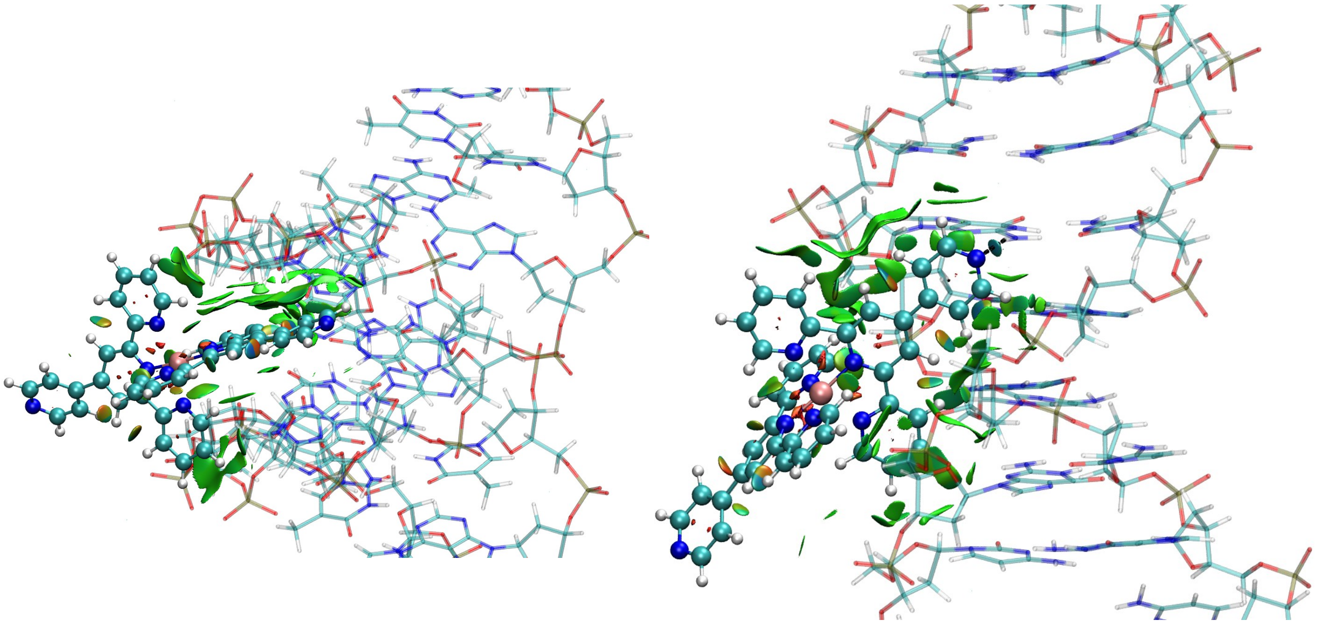

Figure 4: NCI plots for the minor-groove binding of FeC-3 in poly(dA)-poly(dT) (left) and poly(dG)-poly(dC).

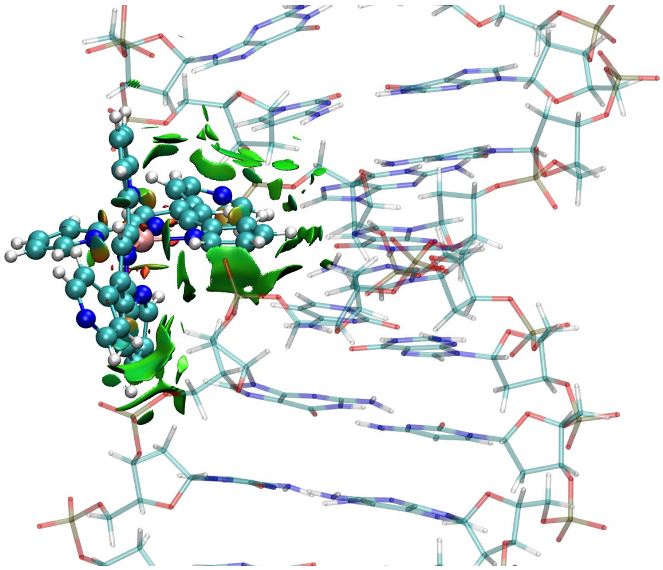

Figure 5: NCI plot for the major groove binding of FeC-3 in poly(dA)-poly(dT)

II) Enthalpy contribution on the binding free energy

	poly(dA)-poly(dT)	poly(dG)-poly(dC)
FeC-1 MinGB	-18.7 +/- 3.1	-17.7 +/- 2.9
FeC-2 MinGB	-27.6 +/- 4.0	-21.7 +/- 3.2
FeC-2 Int	-37.3 +/- 2.8	-36.1 +/- 2.8
FeC-3 MinGB	-28.0 +/- 4.6	-27.6 +/- 5.4
FeC-3 Int	-38.2 +/- 4.5	-38.6 +/- 2.7

Table 1: Enthalpy contribution on the binding free energy of the three complexes in both poly(dA)-poly(dT) and poly(dG)-poly(dC). Results are presented in kcal/mol and were obtained using the MM-GBSA method over 5000 snapshots from the MD.

III) Sketch giving the atom names in the octahedral environment of the iron cation.

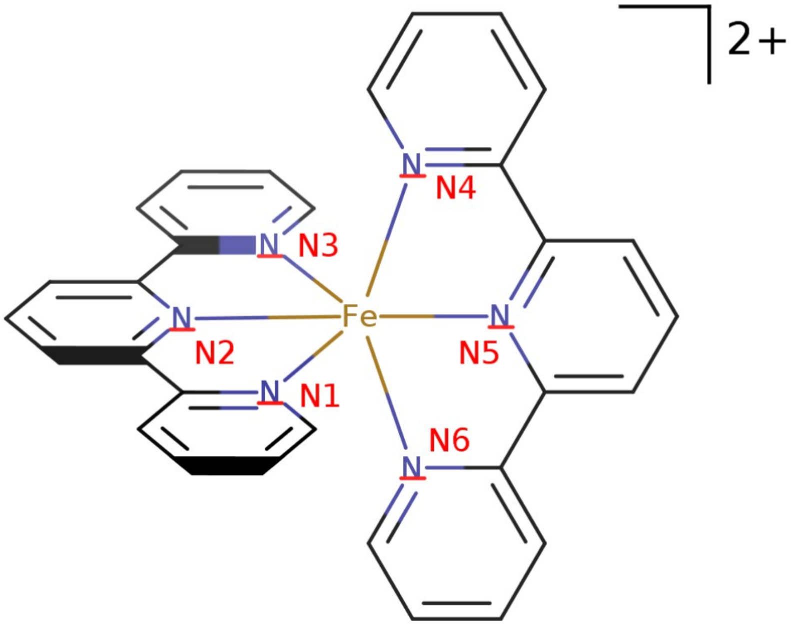

IV) Force field parameters in the octahedral environment of the iron cation.

The force field parameters were fitted manually to respect the octahedral coordinations and the geometry based on the QM geometry optimization. Bond stretching and angle bending were manually imposed to assure only a slight deviation form ideal geometry. Although, this procedures provide a more rigid coordination sphere this is not too much problematic for the following study of DNA interactions since the non-covalent interactions are mostly due to the ligands conjugated systems. Each set of parameters was tested with a 100 ns long MD simulation of the complex in a water box. The geometry variation was compared to the QM equilibrium one. The atoms' point charges were calculated using the classical RESP charges protocol with HF/6-31G* which is recommended for

molecules containing metal atoms.

V) Force field parameters used for FeC-1, FeC-2 and FeC-3. The parameters are given in mol2 file format

MASS
ca 12.010 0.360
ha 1.008 0.135
cp 12.010 0.360
nx 14.010 0.530 same as nb
h4 1.008 0.135
nz 14.010 0.530 same as nb
ny 14.010 0.530 same as nb
nb 14.010 0.530
Fe 55.85 0.600

BOND
ca-ha 344.30 1.087
ca-ca 478.40 1.387
ca-cp 466.10 1.395
cp-nx 486.70 1.340 same as cp-nb
cp-cp 346.50 1.490
nx-ca 483.10 1.342 same as ca-nb
nx-Fe 200.00 2.154
ca-h4 342.90 1.088
cp-nz 486.70 1.340 same as cp-nb
nz-Fe 200.00 2.015
ca-ny 483.10 1.342 same as ca-nb
ny-cp 486.70 1.340 same as cp-nb
ny-Fe 200.00 2.154
cp-nb 486.70 1.340
nb-Fe 200.00 2.015
ca-nb 483.10 1.342

ANGLE
ca-ca-ha 48.460 120.010
ca-ca-ca 67.180 119.970
ca-cp-nx 69.270 121.650 same as ca-cp-nb
ca-cp-cp 64.140 121.140
ha-ca-cp 48.030 121.080
ca-ca-cp 67.240 119.070
cp-nx-ca 68.010 118.040 same as ca-nb-cp
cp-nx-Fe 50.000 119.150
cp-cp-nz 68.050 116.600 same as cp-cp-nb
nx-cp-cp 68.050 116.600 same as cp-cp-nb
nx-ca-ca 69.160 122.630 same as ca-ca-nb
nx-ca-h4 51.820 115.940 same as h4-ca-nb

nx-Fe-nz 50.000 77.750
nx-Fe-nx 50.000 155.500
nx-Fe-ny 50.000 92.570
nx-Fe-nb 50.000 102.270
ca-ca-h4 48.240 121.090
ca-nx-Fe 50.000 119.150
cp-ca-cp 67.180 119.970
cp-nz-cp 68.590 115.860
cp-nz-Fe 50.000 119.150
ca-cp-nz 69.270 121.650 same as ca-cp-nb
ca-cp-ca 67.250 118.330
nz-Fe-ny 50.000 77.750
nz-Fe-nb 50.000 180.000
ca-ny-cp 68.010 118.040 same as ca-nb-cp
ca-ny-Fe 50.000 119.150
h4-ca-ny 51.820 115.940 same as h4-ca-nb
ny-ca-ca 69.160 122.630 same as ca-ca-nb
ny-cp-ca 69.270 121.650 same as ca-cp-nb
ny-cp-cp 68.050 116.600 same as cp-cp-nb
ny-Fe-nb 50.000 77.750
ny-Fe-ny 50.000 155.500 same as ny-Fe-nb
cp-ny-Fe 50.000 119.150
cp-cp-nb 68.050 116.600
cp-nb-cp 68.590 115.860
cp-nb-Fe 50.000 119.150
nb-cp-ca 69.270 121.650
ca-ca-nb 69.160 122.630
ca-nb-ca 68.590 115.860
h4-ca-nb 51.820 115.940

DIHE
ca-ca-ca-ha 1 3.625 180.000 2.000
ca-ca-ca-ca 1 3.625 180.000 2.000
ca-cp-nx-ca 1 4.800 180.000 2.000
ca-cp-nx-Fe 1 4.800 180.000 2.000
ca-cp-cp-ca 1 1.000 180.000 2.000
ca-cp-cp-nz 1 1.000 180.000 2.000
ha-ca-ca-ha 1 3.625 180.000 2.000
ha-ca-cp-nx 1 3.625 180.000 2.000
ha-ca-cp-cp 1 3.625 180.000 2.000
ca-ca-cp-nx 1 3.625 180.000 2.000
ca-ca-cp-cp 1 3.625 180.000 2.000
ca-ca-ca-nx 1 3.625 180.000 2.000
ca-ca-ca-h4 1 3.625 180.000 2.000
ha-ca-ca-cp 1 3.625 180.000 2.000
cp-ca-ca-ca 1 3.625 180.000 2.000
cp-nx-ca-ca 1 4.800 180.000 2.000 same as X -ca-nb-X
cp-nx-ca-h4 1 4.800 180.000 2.000 same as X -ca-nb-X
cp-nx-Fe-nz 1 4.000 180.000 2.000

cp-nx-Fe-nx	1	0.000	000.000	2.000	
cp-nx-Fe-ny	1	4.000	180.000	2.000	
cp-nx-Fe-nb	1	4.000	180.000	2.000	
cp-cp-ca-cp	1	3.625	180.000	2.000	
cp-cp-nz-cp	1	4.800	180.000	2.000	
cp-cp-nz-Fe	1	4.800	180.000	2.000	
nx-cp-cp-ca	1	1.000	180.000	2.000	
nx-cp-cp-nz	1	1.000	180.000	2.000	
nx-ca-ca-ha	1	3.625	180.000	2.000	
nx-Fe-nz-cp	1	4.000	180.000	2.000	
nx-Fe-nx-ca	1	0.000	000.000	2.000	
nx-Fe-ny-ca	1	4.000	180.000	2.000	
nx-Fe-ny-cp	1	4.000	180.000	2.000	
nx-Fe-nb-cp	1	4.000	180.000	2.000	
ca-ca-nx-Fe	1	4.800	180.000	2.000	same as X -ca-nb-X
ha-ca-ca-h4	1	3.625	180.000	2.000	
ca-nx-cp-cp	1	4.800	180.000	2.000	
ca-nx-Fe-nz	1	4.000	180.000	2.000	
ca-nx-Fe-ny	1	4.000	180.000	2.000	
ca-nx-Fe-nb	1	4.000	180.000	2.000	
h4-ca-nx-Fe	1	4.800	180.000	2.000	same as X -ca-nb-X
cp-cp-nx-Fe	1	4.800	180.000	2.000	
cp-ca-cp-ca	1	3.625	180.000	2.000	
cp-nz-cp-ca	1	4.800	180.000	2.000	
cp-nz-Fe-ny	1	4.000	180.000	2.000	
cp-nz-Fe-nb	1	0.000	000.000	2.000	
ca-cp-nz-Fe	1	4.800	180.000	2.000	
ca-cp-ca-ha	1	3.625	180.000	2.000	
ha-ca-cp-nz	1	3.625	180.000	2.000	
nz-cp-ca-cp	1	3.625	180.000	2.000	
nz-Fe-ny-ca	1	4.000	180.000	2.000	
nz-Fe-ny-cp	1	4.000	180.000	2.000	
nz-Fe-nb-cp	1	0.000	000.000	2.000	
cp-ca-ca-h4	1	3.625	180.000	2.000	
cp-ca-ca-nb	1	3.625	180.000	2.000	
ca-ny-cp-ca	1	4.800	180.000	2.000	
ca-ny-cp-cp	1	4.800	180.000	2.000	
ca-ny-Fe-nb	1	4.000	180.000	2.000	
ca-ny-Fe-ny	1	0.000	000.000	2.000	
h4-ca-ny-cp	1	4.800	180.000	2.000	same as X -ca-nb-X
h4-ca-ny-Fe	1	4.800	180.000	2.000	same as X -ca-nb-X
ny-ca-ca-ca	1	3.625	180.000	2.000	
ny-ca-ca-ha	1	3.625	180.000	2.000	
ny-cp-ca-ha	1	3.625	180.000	2.000	
ny-cp-ca-ca	1	3.625	180.000	2.000	
ny-cp-cp-nb	1	1.000	180.000	2.000	
ny-cp-cp-ca	1	1.000	180.000	2.000	
ny-Fe-nb-cp	1	4.000	180.000	2.000	
ny-Fe-ny-cp	1	0.000	000.000	2.000	

cp-ny-ca-ca	1	4.800	180.000	2.000	same as X -ca-nb-X
cp-ny-Fe-nb	1	4.000	180.000	2.000	
cp-cp-nb-cp	1	4.800	180.000	2.000	
cp-cp-nb-Fe	1	4.800	180.000	2.000	
ca-cp-ny-Fe	1	4.800	180.000	2.000	
ca-cp-cp-nb	1	1.000	180.000	2.000	
cp-cp-ny-Fe	1	4.800	180.000	2.000	same as cp-cp-nb-Fe
cp-nb-cp-ca	1	4.800	180.000	2.000	
ca-ca-ny-Fe	1	4.800	180.000	2.000	same as X -ca-nb-X
nb-cp-ca-ha	1	3.625	180.000	2.000	
nb-cp-ca-cp	1	3.625	180.000	2.000	
ca-cp-nb-Fe	1	4.800	180.000	2.000	
ca-cp-ca-ca	1	3.625	180.000	2.000	
ca-ca-nb-ca	1	4.800	180.000	2.000	
ha-ca-ca-nb	1	3.625	180.000	2.000	
ca-nb-ca-h4	1	4.800	180.000	2.000	

IMPROPER

Fe-ca-nx-cp	1.1	180.0	2.0	Using default value
Fe-cp-nz-cp	1.1	180.0	2.0	Using default value
Fe-ca-ny-cp	1.1	180.0	2.0	Using default value

NONBON

ca	1.9080	0.0860	
ha	1.4590	0.0150	
cp	1.9080	0.0860	
nx	1.8240	0.1700	same as nb
h4	1.4090	0.0150	
nz	1.8240	0.1700	same as nb
ny	1.8240	0.1700	same as nb
nb	1.8240	0.1700	
Fe	2.7060	0.4419	

IV) mol2 formated files of FeC-1, FeC-2 and FeC-3 are given for the author convenience.

1) FeC-1

@<TRIPOS>MOLECULE
FE1
59 68 1 0 0
SMALL
No Charge or Current Charge

@<TRIPOS>ATOM

1	C1	2.0400	-3.6620	-0.0610	ca	1	AM1	-0.241001
2	H1	3.0940	-3.9140	-0.0660	ha	1	AM1	0.183266
3	C2	1.0740	-4.6660	-0.0780	ca	1	AM1	0.011763

4 H2	1.3710	-5.7090	-0.0970 ha	1	AM1	0.158688
5 C3	1.6360	-2.3280	-0.0380 cp	1	AM1	0.166931
6 N1	0.3160	-1.9760	-0.0310 nb	1	AM1	0.422442
7 C4	-0.2710	-4.3050	-0.0700 ca	1	AM1	-0.185622
8 H3	-1.0590	-5.0500	-0.0820 ha	1	AM1	0.183170
9 C5	-0.6050	-2.9540	-0.0460 ca	1	AM1	-0.052678
10 H4	-1.6420	-2.6390	-0.0390 h4	1	AM1	0.142980
11 C6	2.5590	-1.1790	-0.0190 cp	1	AM1	-0.049594
12 C7	3.9540	-1.2100	-0.0200 ca	1	AM1	-0.191459
13 H5	4.4910	-2.1510	-0.0350 ha	1	AM1	0.182161
14 N2	1.9010	0.0000	-0.0000 nb	1	AM1	0.738963
15 C8	4.6480	0.0010	-0.0000 ca	1	AM1	-0.042027
16 C9	3.9540	1.2110	0.0190 ca	1	AM1	-0.191373
17 H6	4.4910	2.1520	0.0350 ha	1	AM1	0.182138
18 C10	2.5580	1.1800	0.0190 cp	1	AM1	-0.049802
19 C11	1.6360	2.3280	0.0380 cp	1	AM1	0.167262
20 C12	2.0390	3.6620	0.0610 ca	1	AM1	-0.241140
21 H7	3.0930	3.9140	0.0660 ha	1	AM1	0.183290
22 N3	0.3150	1.9760	0.0310 nb	1	AM1	0.422160
23 C13	1.0730	4.6660	0.0780 ca	1	AM1	0.011834
24 H8	1.3690	5.7090	0.0970 ha	1	AM1	0.158677
25 C14	-0.6060	2.9530	0.0460 ca	1	AM1	-0.052568
26 H9	-1.6420	2.6390	0.0390 h4	1	AM1	0.142996
27 C15	-0.2720	4.3050	0.0700 ca	1	AM1	-0.185695
28 H10	-1.0600	5.0500	0.0820 ha	1	AM1	0.183183
29 C16	0.6050	0.0460	-2.9530 ca	1	AM1	-0.062339
30 H11	1.6420	0.0390	-2.6390 h4	1	AM1	0.146517
31 N4	-0.3160	0.0310	-1.9760 nb	1	AM1	0.444908
32 C17	-1.6360	0.0380	-2.3280 cp	1	AM1	0.148407
33 C18	-2.0400	0.0610	-3.6620 ca	1	AM1	-0.228577
34 H12	-3.0940	0.0660	-3.9140 ha	1	AM1	0.180961
35 C19	-1.0740	0.0780	-4.6660 ca	1	AM1	0.004414
36 H13	-1.3700	0.0970	-5.7090 ha	1	AM1	0.160037
37 C20	-2.5590	0.0190	-1.1790 cp	1	AM1	-0.059901
38 C21	0.2720	0.0700	-4.3050 ca	1	AM1	-0.182474
39 H14	1.0590	0.0820	-5.0500 ha	1	AM1	0.183239
40 N5	-1.9010	-0.0000	0.0000 nb	1	AM1	0.771157
41 C22	-2.5590	-0.0200	1.1800 cp	1	AM1	-0.060168
42 C23	-3.9540	0.0190	-1.2100 ca	1	AM1	-0.186595
43 H15	-4.4910	0.0340	-2.1510 ha	1	AM1	0.182017
44 C24	-3.9540	-0.0200	1.2110 ca	1	AM1	-0.186518
45 H16	-4.4910	-0.0350	2.1520 ha	1	AM1	0.182007
46 C25	-4.6480	-0.0000	0.0000 ca	1	AM1	-0.047372
47 C26	-1.6360	-0.0390	2.3280 cp	1	AM1	0.148798
48 N6	-0.3160	-0.0320	1.9760 nb	1	AM1	0.444717
49 C27	0.6050	-0.0460	2.9530 ca	1	AM1	-0.062164
50 H17	1.6420	-0.0390	2.6390 h4	1	AM1	0.146455
51 C28	-2.0400	-0.0620	3.6620 ca	1	AM1	-0.228811
52 H18	-3.0940	-0.0670	3.9140 ha	1	AM1	0.181012

```
53 C29         0.2720   -0.0700    4.3050 ca        1 AM1      -0.182600
54 H19         1.0590   -0.0820    5.0500 ha        1 AM1       0.183257
55 C30        -1.0730   -0.0780    4.6660 ca        1 AM1       0.004535
56 H20        -1.3700   -0.0970    5.7090 ha        1 AM1       0.160020
@<TRIPOS>BOND
1    1    2 1
2    1    3 ar
3    1    5 ar
4    3    4 1
5    3    7 ar
6    5    6 ar
7    5   11 1
8    6    9 ar
9    7    8 1
10    7    9 ar
11    9   10 1
12   11   12 ar
13   11   14 ar
14   12   13 1
15   12   15 ar
16   14   18 ar
17   15   16 ar
18   15   58 1
19   16   17 1
20   16   18 ar
21   18   19 1
22   19   20 ar
23   19   22 ar
24   20   21 1
25   20   23 ar
26   22   25 ar
27   23   24 1
28   23   27 ar
29   25   26 1
30   25   27 ar
31   27   28 1
32   29   30 1
33   29   31 ar
34   29   38 ar
35   31   32 ar
36   32   33 ar
37   32   37 1
38   33   34 1
39   33   35 ar
40   35   36 1
41   35   38 ar
42   37   40 ar
43   37   42 ar
44   38   39 1
```

```
   45   40   41 ar
   46   41   44 ar
   47   41   47 1
   48   42   43 1
   49   42   46 ar
   50   44   45 1
   51   44   46 ar
   52   46   59 1
   53   47   48 ar
   54   47   51 ar
   55   48   49 ar
   56   49   50 1
   57   49   53 ar
   58   51   52 1
   59   51   55 ar
   60   53   54 1
   61   53   55 ar
   62   55   56 1
   63   57    6 1
   64   57   14 1
   65   57   22 1
   66   57   31 1
   67   57   40 1
   68   57   48 1
@<TRIPOS>SUBSTRUCTURE
     1 AM1        1 TEMP            0 ****  ****    0 ROOT
```

2) FeC-2

```
@<TRIPOS>MOLECULE
FE2
   79    90     1     0     0
SMALL
No Charge or Current Charge


@<TRIPOS>ATOM
      1 C1        -2.0470   -2.8710   -2.2720 ca        1 AM2       -0.260937
      2 H1        -3.1010   -3.0660   -2.4290 ha        1 AM2        0.190549
      3 C2        -1.0820   -3.6600   -2.8940 ca        1 AM2        0.010152
      4 H2        -1.3800   -4.4790   -3.5410 ha        1 AM2        0.154617
      5 C3        -1.6420   -1.8250   -1.4450 cp        1 AM2        0.204612
      6 N1        -0.3210   -1.5500   -1.2260 nx        1 AM2        0.373521
      7 C4         0.2640   -3.3790   -2.6710 ca        1 AM2       -0.188086
      8 H3         1.0500   -3.9650   -3.1330 ha        1 AM2        0.179430
      9 C5         0.5990   -2.3180   -1.8330 ca        1 AM2       -0.046254
     10 H4         1.6360   -2.0720   -1.6390 h4        1 AM2        0.140940
     11 C6        -2.5620   -0.9200   -0.7320 cp        1 AM2       -0.092443
```

12	C7	-3.9540	-0.9450	-0.7490	ca	1	AM2	-0.183853
13	H5	-4.4790	-1.7030	-1.3170	ha	1	AM2	0.152507
14	N2	-1.8980	0.0000	0.0000	nz	1	AM2	0.717132
15	C8	-4.6780	0.0000	-0.0000	cp	1	AM2	0.064982
16	C9	-3.9540	0.9450	0.7490	ca	1	AM2	-0.183854
17	H6	-4.4790	1.7030	1.3170	ha	1	AM2	0.152508
18	C10	-2.5620	0.9200	0.7320	cp	1	AM2	-0.092442
19	C11	-6.1600	0.0000	-0.0000	cp	1	AM2	0.113426
20	C12	-1.6420	1.8250	1.4450	cp	1	AM2	0.204612
21	C13	-2.0470	2.8710	2.2720	ca	1	AM2	-0.260937
22	H7	-3.1010	3.0660	2.4290	ha	1	AM2	0.190549
23	N3	-0.3210	1.5500	1.2260	nx	1	AM2	0.373521
24	C14	-1.0820	3.6600	2.8940	ca	1	AM2	0.010152
25	H8	-1.3800	4.4790	3.5410	ha	1	AM2	0.154617
26	C15	0.5990	2.3180	1.8330	ca	1	AM2	-0.046254
27	H9	1.6360	2.0720	1.6390	h4	1	AM2	0.140940
28	C16	0.2640	3.3790	2.6710	ca	1	AM2	-0.188085
29	H10	1.0500	3.9650	3.1330	ha	1	AM2	0.179430
30	C17	-0.5990	-1.8330	2.3180	ca	1	AM2	-0.052632
31	H11	-1.6360	-1.6390	2.0720	h4	1	AM2	0.141095
32	N4	0.3210	-1.2260	1.5500	ny	1	AM2	0.386120
33	C18	1.6420	-1.4450	1.8250	cp	1	AM2	0.199691
34	C19	2.0470	-2.2720	2.8710	ca	1	AM2	-0.261968
35	H12	3.1010	-2.4290	3.0660	ha	1	AM2	0.191256
36	C20	1.0820	-2.8940	3.6600	ca	1	AM2	0.009645
37	H13	1.3800	-3.5410	4.4790	ha	1	AM2	0.154577
38	C21	2.5620	-0.7320	0.9200	cp	1	AM2	-0.085404
39	C22	-0.2640	-2.6710	3.3790	ca	1	AM2	-0.184977
40	H14	-1.0500	-3.1330	3.9650	ha	1	AM2	0.178728
41	N5	1.8980	0.0000	0.0000	nb	1	AM2	0.701941
42	C23	2.5620	0.7320	-0.9200	cp	1	AM2	-0.085405
43	C24	3.9540	-0.7490	0.9450	ca	1	AM2	-0.185428
44	H15	4.4790	-1.3170	1.7030	ha	1	AM2	0.152703
45	C25	3.9540	0.7490	-0.9450	ca	1	AM2	-0.185426
46	H16	4.4790	1.3170	-1.7030	ha	1	AM2	0.152703
47	C26	4.6780	-0.0000	0.0000	cp	1	AM2	0.066201
48	C27	6.1600	-0.0000	-0.0000	cp	1	AM2	0.115695
49	C28	1.6420	1.4450	-1.8250	cp	1	AM2	0.199691
50	N6	0.3210	1.2260	-1.5500	ny	1	AM2	0.386121
51	C29	-0.5990	1.8330	-2.3180	ca	1	AM2	-0.052633
52	H17	-1.6360	1.6390	-2.0720	h4	1	AM2	0.141095
53	C30	2.0470	2.2720	-2.8710	ca	1	AM2	-0.261968
54	H18	3.1010	2.4290	-3.0660	ha	1	AM2	0.191256
55	C31	-0.2640	2.6710	-3.3790	ca	1	AM2	-0.184977
56	H19	-1.0500	3.1330	-3.9650	ha	1	AM2	0.178728
57	C32	1.0820	2.8940	-3.6600	ca	1	AM2	0.009645
58	H20	1.3800	3.5410	-4.4790	ha	1	AM2	0.154577
59	Fe1	0.0000	0.0000	0.0000	Fe	1	AM2	-2.481741
60	C33	6.8770	-1.1590	0.3470	ca	1	AM2	-0.173910

```
   61 H21        6.3440   -2.0730    0.5920 ha       1 AM2       0.128344
   62 C34        6.8770    1.1590   -0.3470 ca       1 AM2      -0.173911
   63 H22        6.3440    2.0730   -0.5920 ha       1 AM2       0.128344
   64 C35        8.2710    1.1580   -0.3440 ca       1 AM2      -0.087358
   65 H23        8.8080    2.0650   -0.6040 ha       1 AM2       0.139602
   66 C36        8.2710   -1.1580    0.3440 ca       1 AM2      -0.087358
   67 H24        8.8080   -2.0650    0.6040 ha       1 AM2       0.139602
   68 C37        8.9720   -0.0000   -0.0000 ca       1 AM2      -0.083256
   69 C38       -6.8770   -0.3470   -1.1590 ca       1 AM2      -0.172548
   70 H25       -6.3440   -0.5920   -2.0730 ha       1 AM2       0.127726
   71 C39       -6.8770    0.3470    1.1590 ca       1 AM2      -0.172549
   72 H26       -6.3440    0.5920    2.0730 ha       1 AM2       0.127726
   73 C40       -8.2710   -0.3440   -1.1580 ca       1 AM2      -0.084483
   74 H27       -8.8080   -0.6040   -2.0650 ha       1 AM2       0.138728
   75 C41       -8.2710    0.3440    1.1580 ca       1 AM2      -0.084484
   76 H28       -8.8080    0.6040    2.0650 ha       1 AM2       0.138728
   77 C42       -8.9720   -0.0000   -0.0000 ca       1 AM2      -0.087477
   78 H29       10.0580   -0.0000   -0.0000 ha       1 AM2       0.141825
   79 H30      -10.0580   -0.0000   -0.0000 ha       1 AM2       0.142749
@<TRIPOS>BOND
     1     1     2 1
     2     1     3 ar
     3     1     5 ar
     4     3     4 1
     5     3     7 ar
     6     5     6 ar
     7     5    11 1
     8     6     9 ar
     9     7     8 1
    10     7     9 ar
    11     9    10 1
    12    11    12 ar
    13    11    14 ar
    14    12    13 1
    15    12    15 ar
    16    14    18 ar
    17    15    16 ar
    18    15    19 1
    19    16    17 1
    20    16    18 ar
    21    18    20 1
    22    19    69 ar
    23    19    71 ar
    24    20    21 ar
    25    20    23 ar
    26    21    22 1
    27    21    24 ar
    28    23    26 ar
    29    24    25 1
```

30	24	28	ar
31	26	27	1
32	26	28	ar
33	28	29	1
34	30	31	1
35	30	32	ar
36	30	39	ar
37	32	33	ar
38	33	34	ar
39	33	38	1
40	34	35	1
41	34	36	ar
42	36	37	1
43	36	39	ar
44	38	41	ar
45	38	43	ar
46	39	40	1
47	41	42	ar
48	42	45	ar
49	42	49	1
50	43	44	1
51	43	47	ar
52	45	46	1
53	45	47	ar
54	47	48	1
55	48	60	ar
56	48	62	ar
57	49	50	ar
58	49	53	ar
59	50	51	ar
60	51	52	1
61	51	55	ar
62	53	54	1
63	53	57	ar
64	55	56	1
65	55	57	ar
66	57	58	1
67	59	6	1
68	59	14	1
69	59	23	1
70	59	32	1
71	59	41	1
72	59	50	1
73	60	61	1
74	60	66	ar
75	62	63	1
76	62	64	ar
77	64	65	1
78	64	68	ar

```
   79   66   67 1
   80   66   68 ar
   81   68   78 1
   82   69   70 1
   83   69   73 ar
   84   71   72 1
   85   71   75 ar
   86   73   74 1
   87   73   77 ar
   88   75   76 1
   89   75   77 ar
   90   77   79 1
@<TRIPOS>SUBSTRUCTURE
     1 AM2         1 TEMP              0 ****  ****    0 ROOT
```

3) FeC-3

```
@<TRIPOS>MOLECULE
FE3
   77    88     1     0     0
SMALL
No Charge or Current Charge


@<TRIPOS>ATOM
      1 C1          1.3790   -2.7720    2.9780 ca        1 AM3     -0.345863
      2 H1          2.3370   -2.9270    3.4320 ha        1 AM3      0.216300
      3 C2          0.3060   -3.5710    3.3470 ca        1 AM3      0.070885
      4 H2          0.4350   -4.3410    4.0850 ha        1 AM3      0.164434
      5 C3          1.1860   -1.7900    2.0270 cp        1 AM3      0.240247
      6 N1         -0.0080   -1.5910    1.4520 nx        1 AM3      0.144937
      7 C4         -0.9220   -3.3610    2.7540 ca        1 AM3     -0.279161
      8 H3         -1.7790   -3.9540    3.0090 ha        1 AM3      0.205552
      9 C5         -1.0250   -2.3550    1.8090 ca        1 AM3      0.038147
     10 H4         -1.9620   -2.1590    1.3240 h4        1 AM3      0.142512
     11 C6          2.2800   -0.8800    1.5670 cp        1 AM3      0.062030
     12 C7          3.5820   -0.9100    2.0310 ca        1 AM3     -0.336428
     13 H5          3.8960   -1.6240    2.7650 ha        1 AM3      0.197332
     14 N2          1.9110   -0.0040    0.6400 nz        1 AM3      0.424073
     15 C8          4.5100   -0.0090    1.5110 cp        1 AM3      0.148115
     16 C9          4.0860    0.8940    0.5390 ca        1 AM3     -0.337457
     17 H6          4.7810    1.6060    0.1430 ha        1 AM3      0.198160
     18 C10         2.7670    0.8690    0.1250 cp        1 AM3      0.058305
     19 C11         5.9200   -0.0120    1.9840 cp        1 AM3      0.520778
     20 C12         2.1750    1.7830   -0.9000 cp        1 AM3      0.244213
     21 C13         2.9050    2.7640   -1.5420 ca        1 AM3     -0.348288
     22 H7          3.9440    2.9140   -1.3270 ha        1 AM3      0.217132
```

23 N3	0.8750	1.5890	-1.1610	nx	1	AM3	0.148095
24 C14	2.2740	3.5660	-2.4820	ca	1	AM3	0.068124
25 H8	2.8250	4.3340	-2.9920	ha	1	AM3	0.165672
26 C15	0.2810	2.3570	-2.0590	ca	1	AM3	0.033947
27 H9	-0.7600	2.1650	-2.2360	h4	1	AM3	0.143672
28 C16	0.9360	3.3610	-2.7490	ca	1	AM3	-0.276235
29 H10	0.4080	3.9570	-3.4680	ha	1	AM3	0.204945
30 C17	1.1170	-2.0420	-2.1100	ca	1	AM3	0.041940
31 H11	2.0440	-1.8820	-1.5950	h4	1	AM3	0.142177
32 N4	0.0690	-1.3780	-1.6530	ny	1	AM3	0.133495
33 C18	-1.1160	-1.5410	-2.2580	cp	1	AM3	0.250719
34 C19	-1.2670	-2.3810	-3.3440	ca	1	AM3	-0.350310
35 H12	-2.2170	-2.5050	-3.8230	ha	1	AM3	0.217762
36 C20	-0.1620	-3.0740	-3.8180	ca	1	AM3	0.069095
37 H13	-0.2590	-3.7310	-4.6610	ha	1	AM3	0.165322
38 C21	-2.2470	-0.7560	-1.6750	cp	1	AM3	0.052827
39 C22	1.0560	-2.9040	-3.1910	ca	1	AM3	-0.276632
40 H14	1.9360	-3.4200	-3.5230	ha	1	AM3	0.204466
41 N5	-1.9110	0.0040	-0.6400	nb	1	AM3	0.434721
42 C23	-2.8000	0.7660	-0.0160	cp	1	AM3	0.054627
43 C24	-3.5490	-0.7790	-2.1410	ca	1	AM3	-0.332870
44 H15	-3.8280	-1.3750	-2.9860	ha	1	AM3	0.196542
45 C25	-4.1200	0.7950	-0.4290	ca	1	AM3	-0.330056
46 H16	-4.8490	1.3920	0.0780	ha	1	AM3	0.195555
47 C26	-4.5100	0.0090	-1.5110	cp	1	AM3	0.138428
48 C27	-5.9200	0.0120	-1.9840	cp	1	AM3	0.532872
49 C28	-2.2450	1.5480	1.1310	cp	1	AM3	0.243988
50 N6	-0.9360	1.3800	1.3630	ny	1	AM3	0.135167
51 C29	-0.3730	2.0410	2.3600	ca	1	AM3	0.046096
52 H17	0.6770	1.8760	2.5070	h4	1	AM3	0.140948
53 C30	-3.0170	2.3900	1.9080	ca	1	AM3	-0.347610
54 H18	-4.0640	2.5180	1.7180	ha	1	AM3	0.216764
55 C31	-1.0700	2.9040	3.1870	ca	1	AM3	-0.281402
56 H19	-0.5650	3.4170	3.9820	ha	1	AM3	0.205255
57 C32	-2.4180	3.0790	2.9530	ca	1	AM3	0.073040
58 H20	-3.0020	3.7380	3.5690	ha	1	AM3	0.164021
59 Fe1	0.0000	0.0000	0.0000	Fe	1	AM3	-1.555047
60 C33	-6.5730	-1.1740	-2.2900	ca	1	AM3	-0.672422
61 H21	-6.0930	-2.1280	-2.1740	ha	1	AM3	0.225817
62 C34	-6.6210	1.2020	-2.1320	ca	1	AM3	-0.668983
63 H22	-6.1640	2.1540	-1.9340	ha	1	AM3	0.224649
64 C35	-7.9310	1.1450	-2.5810	ca	1	AM3	0.549908
65 H23	-8.4950	2.0500	-2.7170	h4	1	AM3	0.052630
66 C36	-7.8890	-1.1130	-2.7210	ca	1	AM3	0.554795
67 H24	-8.4240	-2.0150	-2.9540	h4	1	AM3	0.050927
68 N7	-8.5520	0.0180	-2.8660	nb	1	AM3	-0.679012
69 C37	6.2190	-0.0960	3.3370	ca	1	AM3	-0.662101
70 H25	5.4480	-0.1380	4.0840	ha	1	AM3	0.223733
71 C38	6.9750	0.0690	1.0850	ca	1	AM3	-0.662618

```
  72 H26        6.8090    0.1120    0.0240 ha       1 AM3      0.224108
  73 C39        7.5510   -0.0890    3.7210 ca       1 AM3      0.544388
  74 H27        7.8140   -0.1410    4.7620 h4       1 AM3      0.053886
  75 C40        8.2690    0.0560    1.5810 ca       1 AM3      0.543134
  76 H28        9.1060    0.1060    0.9090 h4       1 AM3      0.054596
  77 N8         8.5520   -0.0180    2.8660 nb       1 AM3     -0.673510


@<TRIPOS>BOND
     1    1    2 1
     2    1    3 ar
     3    1    5 ar
     4    3    4 1
     5    3    7 ar
     6    5    6 ar
     7    5   11 1
     8    6    9 ar
     9    7    8 1
    10    7    9 ar
    11    9   10 1
    12   11   12 ar
    13   11   14 ar
    14   12   13 1
    15   12   15 ar
    16   14   18 ar
    17   15   16 ar
    18   15   19 1
    19   16   17 1
    20   16   18 ar
    21   18   20 1
    22   19   69 ar
    23   19   71 ar
    24   20   21 ar
    25   20   23 ar
    26   21   22 1
    27   21   24 ar
    28   23   26 ar
    29   24   25 1
    30   24   28 ar
    31   26   27 1
    32   26   28 ar
    33   28   29 1
    34   30   31 1
    35   30   32 ar
    36   30   39 ar
    37   32   33 ar
    38   33   34 ar
    39   33   38 1
    40   34   35 1
```

41	34	36	ar
42	36	37	1
43	36	39	ar
44	38	41	ar
45	38	43	ar
46	39	40	1
47	41	42	ar
48	42	45	ar
49	42	49	1
50	43	44	1
51	43	47	ar
52	45	46	1
53	45	47	ar
54	47	48	1
55	48	60	ar
56	48	62	ar
57	49	50	ar
58	49	53	ar
59	50	51	ar
60	51	52	1
61	51	55	ar
62	53	54	1
63	53	57	ar
64	55	56	1
65	55	57	ar
66	57	58	1
67	59	6	1
68	59	14	1
69	59	23	1
70	59	32	1
71	59	41	1
72	59	50	1
73	60	61	1
74	60	66	ar
75	62	63	1
76	62	64	ar
77	64	65	1
78	64	68	ar
79	66	67	1
80	66	68	ar
81	69	70	1
82	69	73	ar
83	71	72	1
84	71	75	ar
85	73	74	1
86	73	77	ar
87	75	76	1
88	75	77	ar

```
@<TRIPOS>SUBSTRUCTURE
     1 AM3         1 TEMP              0 ****  ****    0 ROOT
```
